# Toward Green Liquid Nitrogen Fertilizer Synthesis: Plasma‐Driven Nitrogen Oxidation and Partial Electrocatalytic Reduction

**DOI:** 10.1002/advs.202411783

**Published:** 2024-12-31

**Authors:** Zhongping Qu, Jungmi Hong, Yuting Gao, Jing Sun, Jingwen Huang, Mingyan Zhang, Mengying Zhu, Tianyu Li, Xiangyu Wang, Dingwei Gan, Qiang Song, Tianqi Zhang, Rusen Zhou, Dingxin Liu, Patrick J. Cullen, Renwu Zhou

**Affiliations:** ^1^ State Key Laboratory of Electrical Insulation and Power Equipment Centre for Plasma Biomedicine Xi'an Jiaotong University Xi'an 710049 P. R. China; ^2^ School of Chemical and Biomolecular Engineering University of Sydney Sydney 2006 Australia

**Keywords:** cost estimation, nitrogen fertilizer, plasma electrocatalytic process, plasma modeling, plasma nitrogen fixation

## Abstract

Liquid fertilizers, particularly when integrated with precision irrigation systems, offer a more efficient and sustainable alternative to traditional solid nitrogen fertilizers. The industrial production of ammonium nitrate (NH_4_NO_3_) is environmentally detrimental due to its reliance on fossil fuels. This study introduces an innovative air‐to‐NOx‐to‐NH_4_NO_3_ pathway for synthesizing liquid nitrogen fertilizer. The process employs an underwater multi‐bubble plasma reactor powered by nanosecond pulse to generate aqueous NOx, which is then partially reduced to NH_4_NO_3_ through electrocatalysis. Results show that the highest NOx production rate, 786.5 mol h^−1^, is achieved when the N_2_/O_2_ ratio closely resemble that of air, and short pulse rise/fall times significantly increase NOx yield. Further plasma diagnostic and global plasma chemistry modeling indicate that short rise/fall times facilitate simultaneous dielectric barrier discharge and spark discharge, synergistically enhancing nitrogen fixation efficiency. The partially electro‐reduced liquid NH_4_NO_3_ fertilizer significantly improves plant growth, with stem length and leaf length increasing by 91.26% and 54.72%, respectively. Cost estimation reveals that 44.22% of the production cost is attributed to electricity consumption, underscoring the potential for optimization with renewable energy integration. Overall, the study provides new insight for the sustainable production and in‐place utilization of liquid nitrogen fertilizers which may advance sustainable agriculture.

## Graphical abstract

This study introduces a method for sustainable synthesis of liquid ammonium nitrate fertilizer using plasma‐electrocatalysis technology, with air and water as the only raw materials. Additionally, it provides an in‐depth exploration of the reaction mechanisms in pulsed discharge plasma, delivering critical scientific insights necessary for optimizing plasma‐electrocatalysis nitrogen fixation and facilitating its practical application.

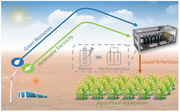

## 1 Introduction

Nitrogen fertilizers are the most widely used chemical fertilizers worldwide, playing a crucial role in boosting food production and sustaining approximately 40% of the global population.^[^ ^1^ ^]^ In traditional agriculture, solid nitrogen fertilizers have been commonly used, mainly manufactured in centralized chemical plants before being transported to agricultural sites.^[^ ^2^ ^]^ Both their production and transportation processes heavily rely on fossil fuels, resulting in substantial greenhouse gas emissions.^[^ ^3^ ^]^ Moreover, when solid nitrogen fertilizers are applied, they inevitably leach into groundwater, leading to a utilization rate of less than 50%.^[^ ^4^ ^]^ Recently, liquid fertilizers combined with precision irrigation systems have been proven to enhance fertilizer utilization rates.^[^ ^5^ ^]^ Therefore, it is of great significance to develop sustainable methods for producing liquid nitrogen fertilizers that can be exploited in a decentralized and on‐demand manner powered by renewable energy.

**Scheme 1:**
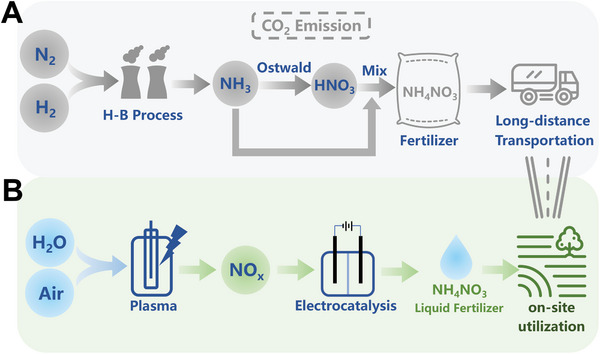
Schematic illustration of A) traditional methods of producing NH_4_NO_3_ and B) the plasma electrocatalytic strategy for sustainable synthesis of liquid NH_4_NO_3_ fertilizer from air and water.

Ammonium nitrate (NH_4_NO_3_) is a widely used liquid nitrogen fertilizer known for its high nitrogen content, with plants able to absorb nitrogen in both ammonium and nitrate forms.^[^ ^6^ ^]^ Additionally, its use in agriculture minimal residue in the soil, making it favored by modern agricultural practitioners. It is established that the abundant nitrogen in air serves as an essential raw material for synthesizing NH_4_NO_3_, involving nitrogen fixation through two key steps: nitrogen oxidation and reduction. Currently, the large‐scale industrial production of NH_4_NO_3_ predominantly relies on the Haber‐Bosch (H‐B) process and the Ostwald process,^[^ ^7^ ^]^ as shown in **Scheme** **1**A. The H‐B process employs a catalytic reaction to convert nitrogen (N_2_) and hydrogen (H_2_) into ammonia (NH_3_) under high temperature and pressure conditions.^[^ ^8^ ^]^ However, the crucial feedstock, molecular hydrogen, is typically generated through energy‐intensive processes such as methane reforming or coal gasification, consuming around 1–2% of the global energy supply and emitting significant amounts of greenhouse gases annually.^[^ ^9^ ^]^ On the other hand, the Ostwald process involves the reaction of ammonia with oxygen at high temperatures and pressures, followed by the absorption of nitrogen oxides with water to produce nitric acid. NH_4_NO_3_ is then synthesized through an acid‐base neutralization process, where NH_3_ reacts with HNO_3_.^[^ ^10^ ^]^ Although both the H‐B and Ostwald processes are well‐established and capable of producing large quantities of NH_4_NO_3_, their stringent reaction conditions restrict them to large‐scale industrial settings. This approach not only results in a significant carbon footprint from the production of solid NH_4_NO_3_ but also increases the risk of explosions during long‐distance transport.

Strategies for sustainable nitrogen fixation at ambient conditions, such as non‐thermal plasma and electrocatalysis, have long been sought to substitute or complement the conventional H‐B process.^[^ ^11^ ^]^ In the plasma‐enabled nitrogen fixation process, plasma discharges can generate energetic electrons through a strong electric field at mild conditions,^[^ ^12^, ^13^ ^]^ activating nitrogen and oxygen molecules and facilitating the formation of nitrogen oxides.^[^ ^14^, ^15^ ^]^ Electrocatalysis, on the other hand, can selectively convert reactants (N_2_ or aqueous NOx) into ammonia by employing suitable catalysts.^[^ ^16^, ^17^, ^18^ ^]^ In recent years, the combination of these two strategies – the plasma‐assisted electrochemical process has been developed,^[^ ^11^, ^19^, ^20^ ^]^ based on the coupling between two fundamental processes: i) plasma‐assisted activation of air to produce NOx in solution; ii) electrochemical conversion of the resultant NOx into ammonium. This hybrid approach offers lower energy consumption and higher product yields compared to these individual methods.^[^ ^11^ ^]^ However, to the best of our knowledge, the direct conversion of air and water into NH_4_NO_3_ as a liquid nitrogen fertilizer using the aforementioned method has rarely been reported.

In this hybrid nitrogen fixation process, a key step is producing NOx effectively and efficiently during the plasma‐enabled nitrogen oxidation process, then favoring the subsequent electrocatalysis. Plasma bubbles have been proven to be an efficient gas–liquid discharge structure in activating N_2_ and O_2_ molecules.^[^ ^21^ ^]^ Introducing high‐density discharges into underwater bubbles can increase the gas‐liquid discharge area and enhance the mass transfer of plasma reactive species, thus contributing to the aqueous NOx production. In recent years, increased focus has been given to plasmas powered by nanosecond pulse sources, because of their advantages of high efficiency and strong reduced electric field, making it easier for the excitation of reactive particles.^[^ ^22^ ^]^ Moreover, the thermal effect of these sources in the processing can be limited due to the extremely short excitation time.^[^ ^23^ ^]^ Although our previous study has demonstrated that the nanosecond‐pulsed plasma bubble discharge system allows for the activation of nitrogen with relatively low energy consumption during the nitrogen fixation process,^[^ ^24^ ^]^ a comprehensive evaluation of the factors affecting the efficiency of nitrogen fixation by bubble discharge has thus far been allusive, together with insightful mechanisms of nitrogen fixation during plasma liquid interactions.

In this study, we demonstrate a tandem plasma‐electrocatalysis integration system for the sustainable synthesis of NH_4_NO_3_ using air and water as raw materials, as illustrated in Scheme 1. The innovative approach combines non‐thermal plasma oxidation and electrocatalytic reduction through an “Air‐to‐NO_x_‐to‐NH_4_NO_3_” pathway. To optimize the nitrogen fixation process and investigate the reaction pathways, we studied the effects of different N_2_/O_2_ ratios of the feed gas, frequencies and pulse rise/fall times of the pulsed power source on nitrogen fixation efficiency, utilizing electrical and optical diagnosis methods, as well as plasma modeling to elucidate the reaction mechanism in the nitrogen fixation process. Electrocatalysis was then introduced to partially reduce the nitrogen fixation products to NH_4_NO_3_. The resulting liquid NH_4_NO_3_ fertilizer was tested for plant cultivation, and a cost estimation was conducted. The results indicate that our liquid nitrogen fertilizer has significant potential for practical applications.

## 2 Results and Discussions

### 2.1 Plasma Assisted Nitrogen Oxidation

#### 2.1.1 Performance of Nitrogen Fixation

**Figure 1:**
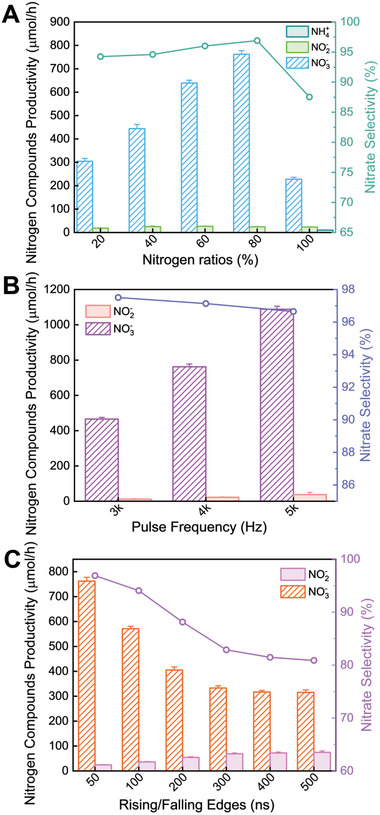
Plasma discharge and product diagnostics for different ratios of N_2_/O_2._ A) Nitrogen compounds productivity of different N_2_/O_2_ ratios. B) Nitrogen compounds productivity of different rise/fall time. C) Nitrogen compounds productivity of different frequencies.

The study first investigated the nitrogen fixation efficiency under different experimental conditions in our plasma reactor setup, focusing on the yields of three nitrogen‐containing compounds. **Figure** **1**A–C shows the concentrations of various nitrogen compounds in the liquid phase after plasma treatment. We initially examined the effect of the feed gas ratio on nitrogen fixation efficiency. The figures demonstrate that under varying N_2_/O_2_ feed ratios, the total nitrogen fixation yield increases as the oxygen ratio decreases, reaching its lowest value when pure nitrogen is used as the discharge gas, with a yield of only 261.3 µmol h^−1^. The optimal nitrogen fixation occurs when the N₂ content is 80% (closely matching the composition of air), achieving a yield of 786.5 µmol h^−1^, which is three times higher than that under pure nitrogen conditions. This suggests a potential correlation between plasma‐assisted nitrogen oxidation pathways or coupled electrocatalytic ammonia synthesis when air is used as the feedstock. The selectivity for NO₃⁻ aligns with the trend of the total nitrogen fixation rate. Notably, NH₄⁺ is detectable even when pure nitrogen is used as the discharge gas, with a yield of 9.9 µmol h^−1^. In the absence of oxygen, nitrogen undergoes disproportionation in the P/L reaction to form ammonia. The results indicate that NO₃⁻ is the main product of liquid‐phase nitrogen fixation under different nitrogen/oxygen ratios. Furthermore, to explore the effects of different discharge power levels on nitrogen fixation, we adjusted the pulse discharge frequencies to 3, 4, and 5 kHz to control the discharge power level. Since the power per pulse remained nearly constant, the power levels for the three groups reached 4.61, 6.14, and 7.68 W, respectively. The effects of different power conditions on nitrogen fixation are shown in Figure 1. Under various frequencies, the liquid‐phase nitrogen fixation products exhibit a linear increase with frequency, reaching 477.74, 786.5, and 1126.2 µmol h^−1^ under low, medium, and high‐power levels, respectively, with NO₃⁻ remaining the main nitrogen fixation product and its selectivity maintained above 96.6%. As our reactor generates plasma discharge at the gas‐liquid interface, it produces a large amount of highly reactive OH radicals, which promote the conversion of low‐valent nitrogen oxides to high‐valent nitrogen oxides (e.g., NO_3_^−^).^[^ ^21^, ^24^ ^]^ Besides, a hybrid mode of DBD and spark discharge generates considerable ozone in the DBD region that further facilitates NO_3_^⁻^ formation in the subsequent spark discharges, resulting in a selectivity of 96.6% for NO_3_^⁻^.^[^ ^14^ ^]^ After determining the optimal nitrogen content and the impact of power on the plasma nitrogen oxide process, we studied the effect of pulse characteristics on plasma nitrogen fixation. The rise/fall time of the pulses may affect the transition of discharge modes, electron energy, and reduced electric field strength, thereby influencing the excitation state of gas molecules and, subsequently, the nitrogen fixation efficiency.^[^ ^25^ ^]^ The results indicate that, similar to other conditions, NO₃⁻ is the primary nitrogen fixation product in the liquid phase. As the pulse rise/fall time decreases, the total nitrogen fixation yield increases, rising from 389.98 to 786.5 µmol h^−1^, nearly doubling. When the pulse rise/fall times are 300, 400, and 500 ns, the differences in total nitrogen fixation yield are minimal. Additionally, the selectivity for NO₂⁻ increases with the pulse rise/fall time. At a pulse rise/fall time of 50 ns, the selectivity for NO₂⁻ is 3.08%, while at 500 ns, this parameter increases to 19.11%.

Additionally, the relationship between the concentration of liquid‐phase nitrogen fixation products and time was measured, as shown in Figures S2–S6 (Supporting Information). It can be observed that, under different conditions, the concentration time curves of total N compounds exhibit an approximately linear relationship. Notably, the change in nitrite concentration is an exception. Except in groups with low nitrogen content, the production of NO_2_^−^ is less related to O_2_, showing an initial rapid increase in concentration, followed by a slower increase, and finally a decrease in concentration.

We speculate that this phenomenon is due to the instability of nitrite in acidic conditions, where, once the concentration reaches a certain level, it is easily oxidized by strong oxidizing species (such as OH) in the solution. At the 20% N_2_ ratio, this trend is not seen for nitrite concentration, likely because the generation rate of NO_2_^−^ is slower and its concentration remains at a lower level.

#### 2.1.2 Mechanisms

To clarify the reaction mechanisms of the plasma nitrogen fixation process and the reasons for the differences in nitrogen fixation efficiency under various conditions, we conducted analyses using both experimental and simulation methods. After the feed gas enters the reactor, it first reacts in the DBD region. Subsequently, the gas activated by the DBD undergoes spark discharge at the gas‐liquid interface, where the resulting nitrogen oxides dissolve in water to form NO₃⁻ and NO₂⁻. To more clearly analyze the process of plasma‐assisted nitrogen fixation, we utilized in situ OES to obtain the relative optical emission intensities from different excited gas species in different energy states within the discharge volume, thereby elucidating the reaction mechanisms. As shown in Figure S7 (Supporting Information), the emission spectra correspond to the feed gas containing 80% N_2_. Prominent N_2_ second positive system (N_2_ (C^3^Πu → B^3^Πg)) lines and the second diffraction signal of the same N_2_(C→B) transition can be observed, as well as the optical emission from excited nitrogen molecular ions (N_2_^+^) at 391.4 nm and weak N atomic lines at 820 nm. Reactive oxygen species, such as O atoms, were also detected at 777.1 nm. The spectra further display the presence of ‐OH (A → X) at 309 nm, Hα at 656 nm, and NO (A → X) at 290 nm.^[^ ^26^, ^27^ ^]^ The presence of these reactive species can effectively explain the nitrogen fixation process: ROS and RNS undergo the Zeldovich reaction and the extended Zeldovich reaction to produce NO.^[^ ^26^, ^28^ ^]^ This NO is subsequently oxidized into higher‐valence nitrogen oxides, which dissolve at the plasma‐liquid interface to form the final products, nitrate and nitrite. Additionally, ozone is formed by the collision of oxygen atoms with O₂ molecules and may play a significant role as an oxidizing agent in the nitrogen fixation process.

**Figure 2:**
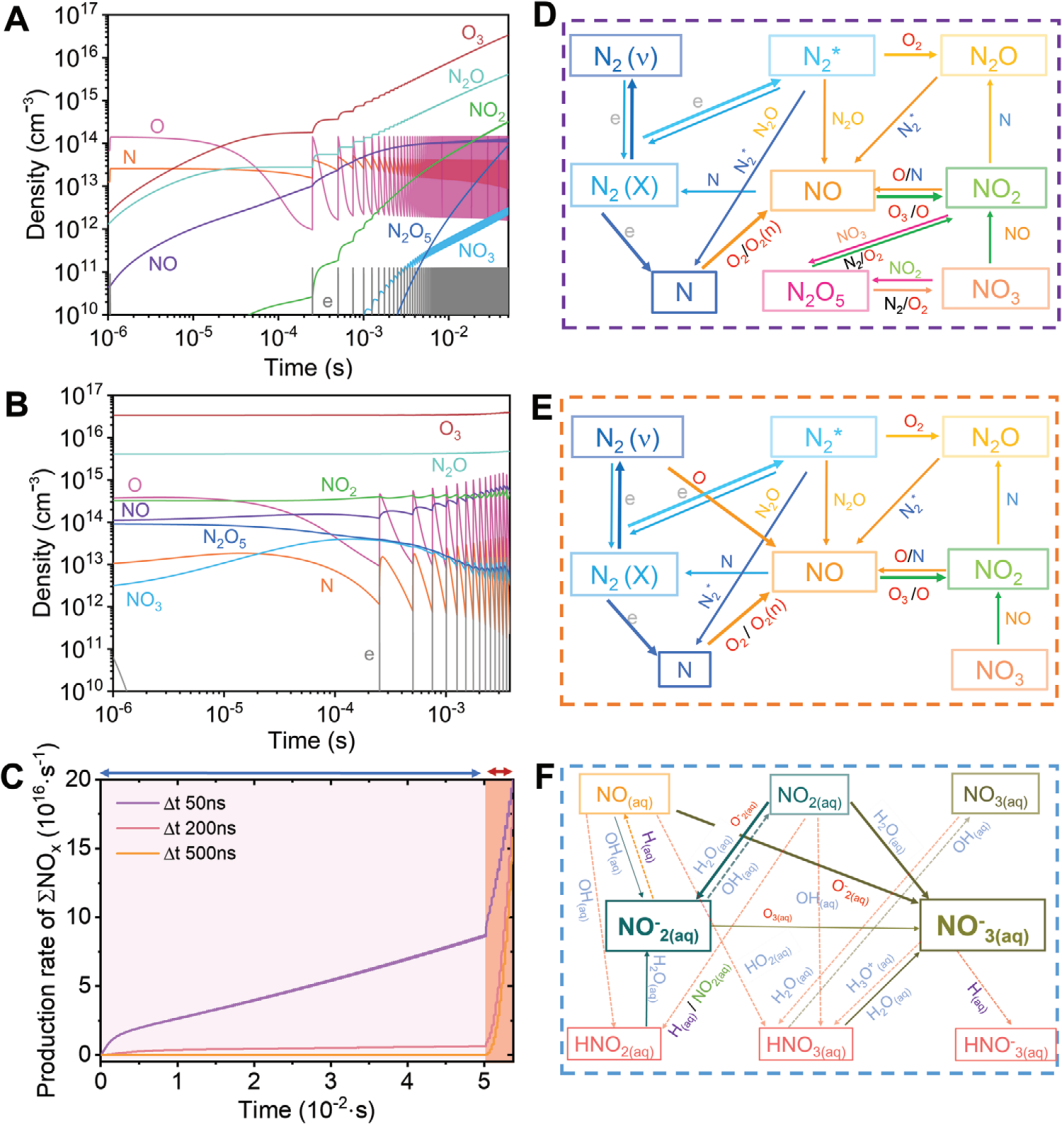
Density profile of important species in A) DBD and B) spark region of N_2_–O_2_ discharge at rising time Δ*t* 50 ns condition. C) Comparison of NOx production rate growth with different plasma condition as a function of rising time of input voltage where the calculated density profile in the unit of cm^−3^ converted into rate (s^−1^) by total discharge volume and residence time compensation. Proposed important chemical pathway for NOx production in D) DBD discharge region and E) spark discharge volume, and F) nitrate and nitrite production in water at Δ*t* 50 ns condition.

To gain a clearer understanding of the chemical reaction processes and pathways during the DBD, spark, and further chemical reactions in water, we performed zero‐dimension plasma chemistry modeling for nitrogen fixation process. Different density profiles of individual NOx and their relevant reactive species are illustrated in **Figure** **2**A,B for both DBD and spark regions under the Δ*t* 50 ns condition. In the DBD discharge, where the gas temperature is low and the electron density is moderate, NO_2_ appears to be the predominant NOx species. In the subsequent spark discharge under lower E/N, higher electron density and gas temperature conditions, NO becomes the major NOx species, consistent with previous studies and literature reports.^[^ ^29^ ^]^ It is also noteworthy that there is a decrease in NOx, N, and O densities during the final electron pulse due to interactions with introduced H_2_O molecules. The strong vibrational‐translational relaxation of N_2_(υ) by ground state H_2_O can result in decreased electron temperatures and reactive species densities, as discussed in earlier works.^[^ ^30^ ^]^ However, it was found that the decrease in NO density was mainly associated with conversion into HNO_2_ through interaction with OH radicals. Additionally, this converted HNO_2_ molecule can readily dissolve in water, transforming into NO_2(aq)_^−^ at a high rate, thereby not constituting a net loss of generated NOx.

The sensitivity analysis of the model was performed to investigate the underlying mechanisms responsible for NOx species formation in both the DBD and spark regions. Figure 2D,E summarizes the key chemical pathways for NOx and related intermediates production in the DBD and spark regions under the Δ*t* 50 ns condition. In the DBD, the predominant NO_2_ species is primarily formed through the oxidation reaction of NO by O_3_ or atomic oxygen, while NO molecules are predominantly produced from the interaction between N atoms and O_2_ or O_2_(υ). Due to the high reduced electric field E/N (230 Td) and lower electron density *n*_e_ (maximum *n*_e_ 1.26 × 10^11^ cm^−3^) with a shorter duration compared to spark discharge, the contribution from N_2_(υ) + O reactions was not significant in the DBD region. In contrast, in the subsequent spark region, lower E/N and higher *n*_e_ conditions (E/N 42 Td, maximum *n*_e_ 1.45 × 10^13^ cm^−3^) lead to significantly increased densities of N_2_(υ) species, making their contribution to NO production noticeable. Additionally, the role of N_2_O*_y_* species becomes minimized at higher gas temperatures during spark discharge.

In the subsequent aqueous phase chemistry, nitrate was observed to have significantly higher densities than nitrite, which supports the measurements. This predominance is primarily due to the interaction of NO_2(aq)_ with H_2_O_(aq)_ as shown in Figure 2F, similar to our previous study.^[^ ^21^, ^24^ ^]^ The contribution from NO_(aq)_ via interaction with O_2(aq)_^−^ was also important for nitrate formation in the combined DBD and spark discharge case at the given plasma condition.

To determine the efficiency of the liquid‐phase absorption process, we conducted FTIR analysis on the exhaust gas after the reaction. The absorption spectra, shown in Figure S8 (Supporting Information), indicate that the main gas‐phase products are O₃ and N₂O, with no detection of other nitrogen oxides (such as NO, NO₂/N₂O₅). This suggests that our reaction setup has a relatively high absorption efficiency.

**Figure 3:**
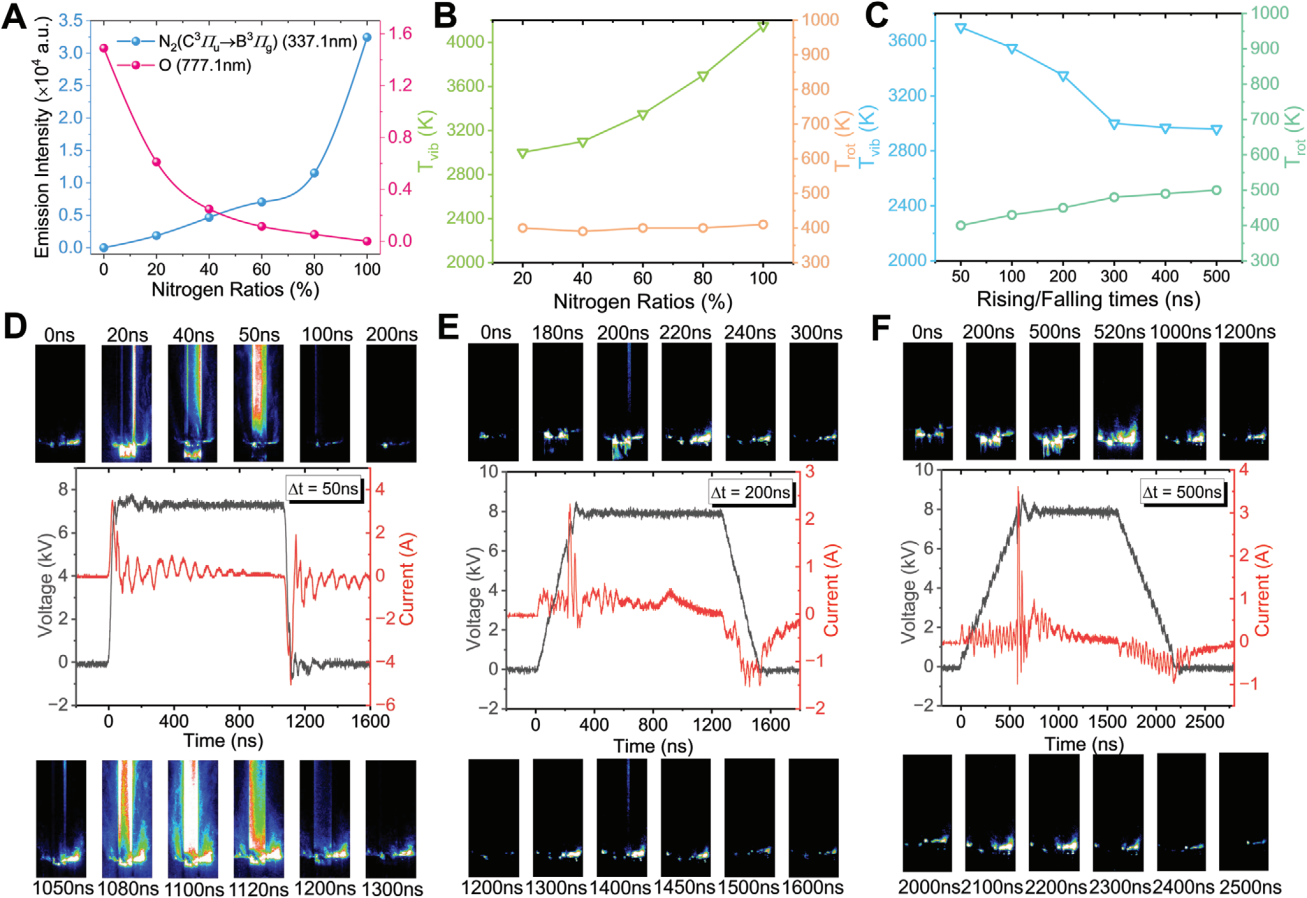
Optical and electrical diagnostic results. A) Comparison of spectral line intensity at 337.1 nm and 777.1 nm for different N_2_/O_2_ ratios. B) Calculated *T*_vib_ and *T*_rot_ for different N_2_/O_2_ ratios. C) Calculated *T*_vib_ and *T*_rot_ for different pulse characteristics. ICCD images and *I*–*V* waveforms of D) 50 ns, E) 200 ns, F) 500 ns.

To study the differences in active species generated in the plasma under different N_2_/O_2_ ratios, we performed OES analysis on the discharge plasma of various feed gases. The spectral images for different N₂/O₂ ratios are shown in Figure S9 (Supporting Information). We compared the intensities of the 337.1 and 777.1 nm spectral lines under different feed gas ratios. These two lines are good indicators of the excitation intensity of activated N₂ molecules and the degree of O₂ dissociation, respectively, as shown in **Figure** **3**A. The results indicate that as the oxygen content increases, the intensity of the 337.1 nm line gradually decreases, while the intensity of the 777.1 nm line gradually increases. A decrease in nitrogen content and an increase in oxygen content are expected to reduce collisions between high‐energy electrons and N₂ molecules, thereby reducing the formation of RNS.^[^ ^31^, ^32^ ^]^ This reduction is reflected in the emission spectra as a significant decrease in the intensity of the N₂ lines and their associated diffraction lines.

Additionally, gas temperature is a key physical parameter in plasma discharge processes, influencing plasma reactivity. The vibrational temperature *T*_vib_, is to be influenced by electron temperature and vibrational energy transfer process, indicates the energy of various molecular excited states, while *T*_rot_ reflects the macroscopic temperature of the plasma.^[^ ^33^ ^]^ Calculating *T*_vib_ is essential for studying the reaction characteristics of plasma discharges with different pulse voltage rise and fall times. The rotational energy level distribution of excited diatomic molecules can be obtained through OES to calculate averaged gas temperature.^[^ ^34^, ^35^ ^]^ *T*_vib_ and *T*_rot_ of nitrogen in different N_2_/O_2_ ratios were calculated (Figure 3B). The results show that the vibrational temperature of nitrogen increases with the concentration of nitrogen, reaching 4150 K under pure nitrogen conditions, while the rotational temperature remains almost unaffected by the gas ratio. A higher vibrational temperature indicates greater reactivity of nitrogen. These two phenomena can explain the differences in nitrogen fixation efficiency with different feed gases: oxygen is an electronegative gas, and changes in its concentration can affect the interaction patterns and efficiency of electrons within the plasma. This, in turn, influences the generation of various active nitrogen and oxygen species, leading to changes in plasma‐liquid (P/L) reactions, and ultimately impacting nitrogen fixation outcomes. When discharging pure nitrogen gas, only ∙OH acts as an oxidant, naturally reducing the production of nitrogen oxides. Increasing the oxygen content introduces more ROS, which leads to the oxidation of nitrogen into nitrogen oxides. However, an excessive increase in oxygen, being an electronegative gas, absorbs high‐energy electrons, preventing nitrogen from being sufficiently excited to participate in reactions. This is reflected in the decrease in vibrational temperature, ultimately reducing the yield.

To investigate how different pulse characteristics affect discharge properties and thereby influence nitrogen fixation efficiency, we collected ICCD images and current‐voltage waveforms (Figure 3D–F). It can be observed that the current shows a single pulse during the pulse rise and fall times under different pulse voltages. However, as the fall time increases, the reverse pulse current during voltage fall becomes weaker compared to the forward pulse current during voltage rise, and the width of the pulse current increases with the fall time. Images captured by the ICCD camera show that during the nitrogen fixation process, both DBD and spark discharge occur simultaneously within the reactor. At the gas‐liquid interface, RNS and ROS in the bubbles transfer into the solution and are absorbed. While the discharge intensity decreases with the pulse voltage rise/fall, consistent with previous nitrogen fixation results. Notably, at a pulse voltage rise/fall time of 50 ns, in addition to spark discharge, a significant DBD can be observed when the pulse current reaches its peak, with DBD intensity during voltage fall significantly higher than during voltage rise. Conversely, at a rise/fall time of 200 ns, only weak DBD can be observed. At a rise/fall time of 500 ns, only spark discharge can be observed. Thus, an increase in pulse voltage rise/fall time is associated with a decrease in DBD intensity. This phenomenon may be due to the spark discharge being more likely to occur at the gas‐liquid interface, causing bubble rupture. When the pulse voltage rise/fall time is short, the space electric field changes rapidly, promoting rapid charge accumulation and forming a DBD between the electrode and quartz tube. Conversely, with longer rise/fall times, charge accumulation is slower, making discharge more likely at the gas‐liquid interface at the pores, favoring spark discharge paths over DBD. The DBD reactor can be equated to a capacitor, where the rate of change of voltage dv/dt is greater with shorter pulse rise/fall times, resulting in larger capacitive currents. Conversely, with longer pulse rise/fall times, the rate of voltage change is smaller, resulting in smaller capacitive currents. This explanation is reflected in the plasma, where longer pulse rise/fall times lead to smaller and less intense DBD. Therefore, this result indicates that the discharge mode can be adjusted by changing pulse parameters. We believe this result explains the increased selectivity for nitrite (NO₂⁻) with longer pulse rise/fall times. Spark discharge generates a significant amount of NO (as discussed later), which subsequently reacts with hydroxyl radicals (∙OH) to form NO₂⁻, the primary source of nitrite in the solution. As the pulse rise/fall time increases, the relative intensity of spark discharge also strengthens, resulting in a higher proportion of NO₂⁻ production. This is the main factor contributing to the enhanced selectivity toward nitrite. This phenomenon further supports the reasons for the high nitrate selectivity in our system. The weakening of DBD with increasing pulse rise/fall times, alongside the increase in nitrate selectivity, demonstrates the crucial role of DBD in achieving high nitrate selectivity.

The nitrogen temperatures for different pulse rise/fall times were also calculated, as shown in Figure 3C. As the pulse voltage rise/fall time increases, the gas vibrational temperature decreases, indicating that the reactivity of excited nitrogen in the plasma decreases continuously. Combining the ICCD images in Figure 3, it can be observed that the discharge intensity decreases with increasing pulse voltage rise/fall time. This means the energy gained by electrons decreases, leading to less energy transferred to N_2_ molecules through collisions, resulting in a lower nitrogen vibrational temperature. This phenomenon can explain the decrease in nitrogen fixation yield with increased pulse rise/fall time, as a lower vibrational temperature indicates a smaller average number of reactive particles involved in oxidation reactions, leading to a lower final yield. On the other hand, plasma power can be calculated based on current–voltage images, showing a slight increase in pulse power with increasing pulse rise/fall time, resulting in an increase in the macroscopic temperature *T*_rot_ of the reactive gas with increasing pulse rise/fall time.

To gain a deeper understanding of the changes in discharge modes and the differences in the generation of active species caused by different pulse rise/fall times, we utilized modeling to analyze the underlying mechanisms. Figure 2F illustrates the temporal profiles of total NOx species production, including NO, NO_2_, NO_3_, N_2_O_3_, N_2_O_4_, N_2_O_5_, and HNOx species, under different conditions of estimated reduced electric field (E/N) and electron density as discussed in Figure S12 (Supporting Information). For a fast‐rising voltage input (Δ*t* 50 ns), as discussed previously, both high‐intensity DBD and spark discharges were observed in the experiment. The model showed a gradual increase in NOx production in the DBD over a relatively longer residence time of *τ* ≈ 50 ms compared to the spark discharge (*τ* ≈ 3.71 ms). Subsequent spark discharge further accelerated NOx production, reaching a maximum rate of 2.0 × 10^16^ s^−1^ in the gas phase under Δ*t* 50 ns conditions, which was approximately 2.4 times higher than the DBD alone.

In contrast, for the Δ*t* 500 ns rising time condition, the DBD discharge was suppressed, and only spark discharge was observable. Total power dissipation occurred within the confined discharge volume of the spark region, facilitating rapid NOx production. However, the overall production rate of total NOx was 1.3 times higher under the Δ*t* 50 ns conditions where a combined DBD and spark discharge was present compared to the spark only discharge under Δ*t* 500 ns.

A further comparison of the N₂(υ) density and distribution between the Δ*t* = 50 ns and Δ*t* = 500 ns conditions, as shown in Figure S13 (Supporting Information), confirmed a higher total density of N₂(υ) and a greater ratio of high‐lying N₂(υ) species. This supports the observation of a higher *T*_vib_ in the Δ*t* = 50 ns condition, which is attributed to the higher V–V interaction rate resulting from the transfer of N₂(υ) species from the DBD region prior to the spark discharge.

It is considered that the transfer of high‐density reactive species including N₂(υ) species from the DBD region was advantageous in enabling higher production rates within the limited discharge volume and residence time of the spark discharge, even at lower power levels (SEI 0.404 J cm^−3^) compared to the Δ*t* 500 ns condition (SEI 0.414 J cm^−3^). However, it is worth noting that increasing the volume of the spark discharge or raising the power level may enhance NOx production independently, leveraging the benefits of high‐density electrons and elevated gas temperatures. Therefore, the synergistic enhancement effect observed with combined DBD and spark discharge appears to be dependent on a specific power range. Given the current electrode configuration and moderate power levels, the combination of DBD and spark discharges has been confirmed as important for improving the NOx production rate in both the model and experimental observations. The temporal profiles of NOx species under the Δ*t* 500 ns condition and the pathway differences for the Δ*t* 500 ns condition are discussed in detail in Figure S14 in the Supporting Information.

### 2.2 N Fertilizer Synthesis and Planting Applications

**Figure 4:**
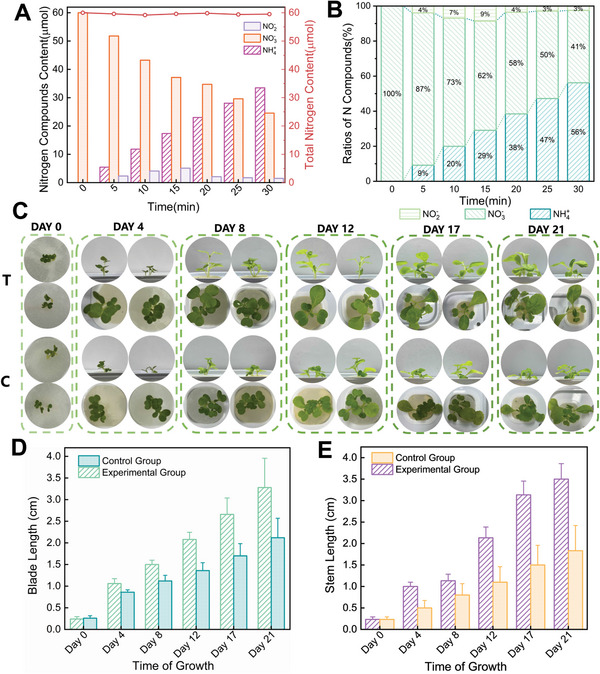
Electrocatalytic reduction of NOx to produce liquid NH_4_NO_3_ fertilizer for application. A) The amount of NH_4_^+^, NO_3_^−^, NO_2_^−^ and total N. B) The change of the percentage of different N‐containing compounds with the reduction process. C) Comparison of growth state between fertilizer‐applied plants and controls. D) Comparison of blade length. E) Comparison of stem length.

In the plasma oxidation nitrogen fixation section, we report that using air as the reaction gas and employing short pulse rise/fall times resulted in better nitrogen fixation and higher nitrate selectivity. Under these conditions, the nitrogen fixation product can be considered to consist solely of nitrate. In the electrocatalytic process, the nitrate, nitrite, and ammonium ion concentrations in the electrolyte were measured, as shown in **Figure** **4**A. During the electrolysis process, nitrate is continuously consumed as the reactant, while ammonium ions are steadily produced as the product, with a relatively constant yield. Nitrite acts both as a reactant and a product in the electrolysis cell, showing an initial increase followed by a decrease in its concentration in the electrolyte as the electrolysis time progresses.

From 0 to 15 min of electrolysis, the nitrate concentration is relatively high, allowing two reduction reactions to occur simultaneously at a given reduction potential, producing nitrite and ammonium ions. The concentration of substances in the liquid phase indicates that the rate of nitrite reduction to ammonia is slower than the rate of nitrite formation, leading to an increase in nitrite concentration. Between 15 and 20 min, the nitrite concentration reaches its peak. During this period, the nitrate reduction rate decreases compared to the previous stage, while the nitrite concentration rapidly decreases, indicating that nitrite reduction becomes the dominant reaction at the cathode. After 20 min of electrolysis, the nitrite concentration stabilizes and reaches dynamic equilibrium, with the primary cathode reaction being the conversion of nitrate to ammonium ions. Additionally, it is worth noting that the total nitrogen content remains almost unchanged, suggesting that the initial nitrate is not reduced to nitrogen gas. When the concentrations of nitrate and ammonium ions reach equilibrium, the synthesis of ammonium nitrate can be considered successful. According to the measurements of the liquid phase concentrations, ammonium nitrate is presumed to have formed in the solution between 25 and 30 min of electrolysis.

To verify the effectiveness of our plasma electrocatalytic ammonium nitrate fertilizer, we conducted a hydroponic experiment using Chinese cabbage. We found that the growth rate of the fertilized group was significantly faster, and the differences in leaf size and stem length between the fertilized and unfertilized plants became increasingly pronounced over time. On day 21, the stem length and leaf length of the experimental group reached 3.5 cm and 3.28 cm, respectively, compared to 1.83 cm and 2.12 cm in the control group, representing increases of approximately 91.26% in stem length and 54.72% in leaf length. The experimental group's plants exhibited faster growth, with larger and more luxuriant leaves, as evident from the growth photos. Additionally, when comparing the growth states of the two groups at 17 and 21 d, the control group's leaves appeared duller with yellowing edges, while the experimental group maintained vibrant green leaves. This may be because our liquid fertilizer provided the necessary nitrogen (N) for leaf development, whereas the control group, lacking sufficient N over time, exhibited leaf yellowing. These results indicate that the liquid ammonium nitrate fertilizer produced in our study has a positive effect on agricultural plant growth.

Additionally, to compare the effects of the ammonium nitrate liquid fertilizer produced by our process with those of commercial ammonium nitrate fertilizer on plant growth, we prepared a commercial ammonium nitrate solution with the same concentration as the experimental solution and conducted a comparative plant growth experiment. Photographs of the three groups of cabbage, along with stem length and leaf length statistics, are presented in Figures S19–S21 (Supporting Information). The results demonstrate that the ammonium nitrate solution produced by our process effectively promotes plant growth, with performance comparable to commercial ammonium nitrate fertilizer at the same concentration.

### 2.3 Cost Estimation

**Figure 5:**
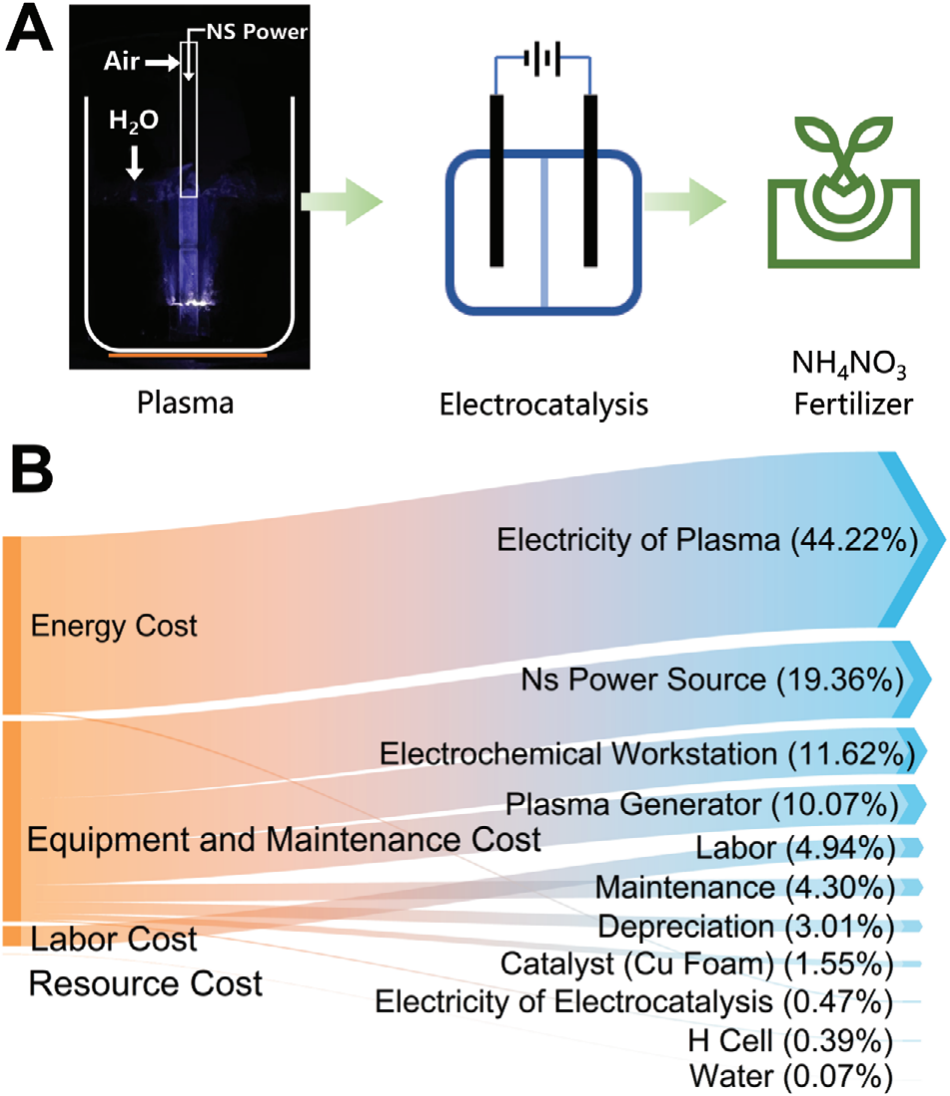
A) Schematic of the sustainable synthesis of liquid fertilizer by plasma electrocatalysis. B) Cost estimation of the synthesis process.

To determine the feasibility of the plasma electrocatalytic technology for liquid ammonium nitrate fertilizer production proposed in this study, we conducted a cost estimation. The estimation utilized energy consumption data for ammonium nitrate synthesis from this study, along with other reasonable assumptions (see Supporting Information). The energy consumption for plasma generation of NOx in the liquid phase was 24.8 MJ mol^−1^, and the Faradaic efficiency of the Cu foam electrocatalytic reduction of nitrogen oxides to ammonium was 60%. Our estimation model accounted for four cost components: energy cost, equipment and maintenance cost, labor cost, and resource cost. As shown in **Figure** **5**B, energy costs, particularly for plasma oxidation, comprised a significant portion of the total cost (44.24%), while the energy cost for electrocatalysis was only 0.47% of the total. The cost of setting up production lines also constituted a substantial proportion. It is foreseeable that integrating renewable energy sources will significantly reduce energy costs, thereby lowering overall production costs. Our plasma electrocatalytic ammonium nitrate synthesis technology demonstrates application prospects and potential.

The combined Haber‐Bosch and Ostwald processes have achieved an energy consumption of 0.6 MJ mol^−1^, approaching the theoretical limit. This energy consumption is still far lower than that of nitrogen fixation through plasma‐electrocatalysis coupling.^[^ ^36^ ^]^ However, according to our investigation, in China, the wholesale price for 1 ton of 1 mm ammonium nitrate liquid fertilizer is approximately $769.2 (Henan Tianyun Chemical Co., Ltd., https://shop6804986935c97.1688.com/). In comparison, our cost estimation shows that producing the same concentration and quantity using our method would cost only $2.06. Although the market price includes profit margins, the several‐hundred‐fold price difference indicates that our technology offers potential economic benefits compared to traditional methods. The production of liquid nitrogen fertilizers in industry involves a dissolution process, which requires additional equipment. The added cost of these devices and the increased energy consumption may contribute to the higher price of industrially produced liquid nitrogen fertilizers.

Additionally, the H‐B process, as the most mature and large‐scale nitrogen fixation method, has been optimized over hundreds of years to approach its theoretical energy efficiency limit. Our proposed method is not intended to replace or challenge the combined Haber‐Bosch and Ostwald processes in terms of energy efficiency or cost. Instead, it serves as a complementary approach to traditional methods, offering the advantages of distributed, small‐scale production with a focus on green and sustainable practices of liquid nitrogen fertilizers. Moreover, it provides a potential pathway for integrating and utilizing renewable energy sources.

## 3 Conclusion

In this study, we successfully synthesized liquid ammonium nitrate fertilizer under mild, miniaturized experimental conditions using a novel plasma‐assisted cascade electrocatalytic method. Our research showed that a 20% oxygen ratio can optimize nitrogen fixation performance by increasing ROS content and minimizing competition for N2 activation. Additionally, altering plasma power levels through frequency changes did not affect nitrogen fixation energy consumption. Combining experiments and simulations, we found that the rise/fall rate of pulses can alter the relative intensity of DBD and spark discharges. Shorter rise/fall times result in higher vibrational temperatures and yields. 0D plasma chemistry modeling demonstrated the enhanced effect of coupling DBD and spark discharge at similar power levels. Under rapidly rising pulse conditions, effective DBD discharge can be achieved before spark discharge, providing high‐density reactive species (including vibrationally excited molecules) to enhance NOx formation within the short residence time of the spark discharge. Electrocatalysis can partially reduce NO_3_^−^ to synthesize NH_4_NO_3_ within a certain time. The synthesized liquid fertilizer was used for cabbage growth, improving growth rates for approximately 55% and promoting lush foliage. Finally, the cost estimation indicates that the synthesis of liquid ammonium nitrate fertilizer using this technology is significantly cheaper than the market price. Notably, over 44% of the total cost is attributed to electricity consumption, suggesting that the use of renewable energy could further reduce costs. This demonstrates the promising potential of this method for widespread application.

## Conflict of Interest

Authors P.C. and T.Z. are associated with PlasmaLeap Technologies, the supplier of the plasma bubble technology employed in the study. Authors P.C., T.Z., and R.Z. are listed as inventors of patent application WO2022/073071 A1 “Plasma assisted electrocatalytic conversion.”

## Supporting information



Supporting Information

## Data Availability

The data that support the findings of this study are available from the corresponding author upon reasonable request.
