# Supplementary material for: Toward Green Liquid Nitrogen Fertilizer Synthesis: Plasma‐Driven Nitrogen Oxidation and Partial Electrocatalytic Reduction
Source: Adv Sci (Weinh). 2024 Dec 31;12(8):2411783. doi: 10.1002/advs.202411783 (PMC11848547; doi:10.1002/advs.202411783)
Supplement: Supplementary file 1 — Supporting Information [file ADVS-12-2411783-s001.docx]

Supporting Information

Towards Green Liquid Nitrogen Fertilizer Synthesis: Plasma-Driven Nitrogen Oxidation and Partial Electrocatalytic Reduction

Zhongping Qu, Jungmi Hong, Yuting Gao, Jing Sun, Jingwen Huang, Mingyan Zhang, Mengying Zhu, Tianyu Li, Xiangyu Wang, Dingwei Gan, Qiang Song, Tianqi Zhang, Rusen Zhou, Dingxin Liu, Patrick J. Cullen, Renwu Zhou^*^

**1. Experimental Setup**

**1.1. Plasma Reactor System**

The experimental setup is illustrated in Figure S1. The plasma is driven by a pulse power supply (Smart Maple HV-2015, China). The plasma multi-bubble reactor consists of a cylindrical quartz tube and a stainless steel high-voltage electrode. The bottom of the outer quartz tube is evenly distributed with 8 micropores, which serve as gas outlets and plasma discharge channels. The reactor is placed within a beaker, and the bottom of the beaker is affixed with copper foil serving as the ground electrode. The beaker contains 150 mL of dilute H_2_SO_4_ solution (pH = 2) as the liquid phase, intended to absorb gas-phase products during the reaction and maintain stable electrical conductivity and pH value throughout the discharge process. The default pulse power supply parameters are set at 4 kHz with a pulse voltage rise/fall time of 50 ns, and the pulse width is set at 1 µs. The default feed gas is a mixture of N_2_ and O_2_, with nitrogen comprising 80% of the gas mixture. The total feed gas flow rate is controlled at 1 L/min by a mass flow controller (Sevenstar D07-26).


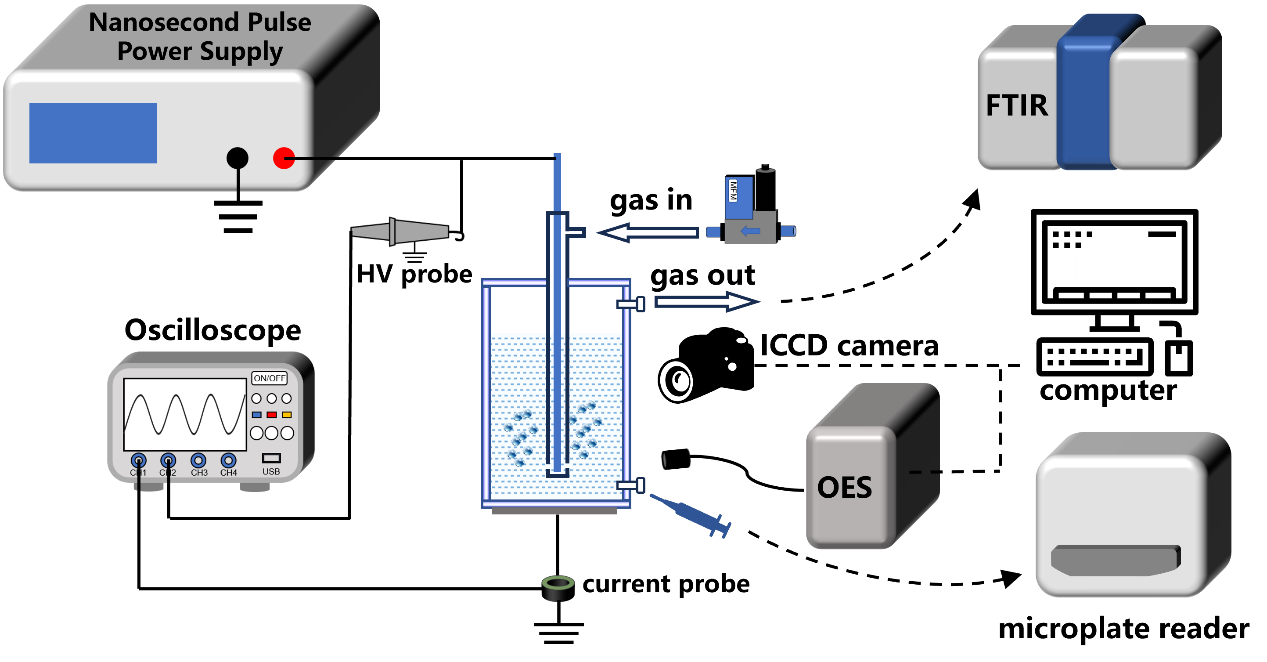


**Figure. S1** Schematic diagram of the experimental system

**1.2 Optical and electrical Diagnostics**

A digital oscilloscope (Tektronix, DPO3052) measures current and voltage data and displays waveform graphs, connected to a high-voltage probe (Tektronix P6015A) and a current probe (Tektronix, P6139A). An ICCD camera (PI-MAX3; Princeton Instruments) captured discharge intensity during the discharge process, positioned in front of the reactor. The OES diagnostic equipment employs an Andor SR750i monochromator (grating 1200 lines mm^−1^) with a spectral range set between 200-850 nm. The discharge photographs were taken with a digital camera (Nikon D7000) with an exposure time of 1s.

The plasma power consumption was calculated using the current and voltage data collected through an oscilloscope. The specific calculation method is outlined as follows.

In the equation, *T* represents the duration of a single pulse, *u* and *i* represent the plasma voltage and current within a single pulse, and *f* is the pulse repetition frequency.

**1.3 Electrochemical System**

The electrochemical workstation was a CHI Potentiostat (CHI 760E), and the electrocatalytic process was carried out in an H-cell. The cathode and anode compartments were separated by a proton exchange membrane. A three-electrode electrochemical configuration was utilized, comprising the working electrode (WE), platinum wire counter electrode (CE), and Ag/AgCl reference electrode. The WE was equipped with Cu foam as the electrocatalytic material. To facilitate electrochemical control over different concentrations, a prepared solution of sodium nitrate with a pH of 2 replaced plasma-activated water as the electrolyte containing nitrate ions, with 30 mL of solution placed in the cathode chamber. The LSV curve is shown in Figure S10. Ionic exchange was facilitated by a magnetic stirrer operating at 650 rpm during the electrochemical reaction. During the electrocatalytic reduction of NO₃⁻, the reduction potential is -0.5 V vs. RHE, with a current density of -25.4 mA/cm² and a Faradaic efficiency of 59.9%.

**1.4 Product detection and analysis**

Long-lived nitrogen-containing compounds in the liquid phase may include NO_3_^-^, NO_2_^-^, and NH_4_^+^. Quantitative measurements of these reactive species are conducted using colorimetric methods. Reagents employed for this purpose include the Nitrate/Nitrite Colorimetric Assay Kit (Cayman, 780001, USA) and the Griess reagent Kit (Beyotime, S0021, China). The FTIR diagnostic instrument (Bruker, Tensor II) detects gas-phase products, connected to the gas outlet after passing through water.

**1.4.1. Nitrite Measurement Method**

Add plasma activated water or diluted activated water to Griess reagent R1 and Griess reagent R2. After color development, measure the absorbance at 540 nm using a spectrophotometer. Prepare standard solutions and their dilutions, and determine the relationship between the absorbance of NO₂⁻ at 540 nm and its concentration by the same method, then plot the standard calibration curve. Compare the absorbance of the processed plasma samples with the standard curve to determine the NO₂⁻ concentration in the samples.

**1.4.2. Nitrate Measurement Method**

Add plasma-activated water (pH adjusted to 7 to minimize the effect of low pH on NO₃⁻ reduction and prevent measurement errors), enzyme co-factors, and nitrate reductase mixture. Incubate in the dark at room temperature for one hour. Then, add Griess reagent 1 and Griess reagent 2. After color development, measure the absorbance at 540 nm using a spectrophotometer. Prepare gradient dilutions of a standard solution containing known concentrations of NO₂⁻ and NO₃⁻ and plot a standard calibration curve using the same method. Compare the absorbance of the processed samples with the standard curve to determine the total concentration of NO₃⁻ and NO₂⁻ in the samples. Subtract the measured NO₂⁻ concentration to obtain the NO₃⁻ concentration in the samples.

**1.4.3. Ammonia (NH_4_^+^) Measurement Method**

Take the sample to be tested and add 1 M NaOH solution (containing 5 wt% salicylic acid and 5 wt% sodium citrate), 0.05 M NaClO, and 1 wt% C₅FeN₆ Na₂O aqueous solution. Incubate the mixture in the dark at room temperature for one hour, then measure the absorbance at 655 nm. Prepare gradient dilutions of a standard solution with known NH₄⁺ concentrations and plot a standard calibration curve using the same method. Compare the absorbance of the sample with the standard curve to determine the total concentration of NH₄⁺ in the sample.

**1.5 Processes of growing plants**

Rapidly maturing Chinese cabbage seeds used in the experiment were purchased from Ideal Agriculture Company, China. After germination, the seeds were cultivated in hydroponic boxes, each containing 350 mL of deionized water, maintaining a constant temperature of 26 °C with 24-hour light exposure. The plants were divided into four groups, with six lettuce seedlings in each group. Two groups served as control groups, while the other two were experimental groups. Every five days, the control groups received 2 mL of 0.2% K_2_SO_4_ solution and 10 mL of ultrapure water. The experimental groups received 2 mL of 0.2% K_2_SO_4_ solution and 10 mL of laboratory-prepared ammonium nitrate solution every five days. Photographs of the plant growth process were taken, and the stem lengths of the lettuce seedlings were measured. The planting procedure should be repeated three times to ensure accuracy and reliability of the results.

**1.6 Statistical Analysis**

Statistical analysis and graphing were carried out using OriginPro. Data are from several independent experiments and the experimental results were presented as mean ± standard deviation (SD). Statistical analysis was performed using student's t-test.

**2. Experimental results**





**Figure. S2** Variation of NOx concentration with time for different N_2_/O_2_ ratios

**

**

**Figure. S3** Variation of NO_2_^-^ concentration with time for different N_2_/O_2_ ratios





**Figure. S4** Variation of NH_4_^+^ concentration with time in pure N_2_ condition





**Figure. S5** Variation of NOx concentration with time for different frequencies





**Figure. S6** Variation of NOx concentration with time for different rising/falling times


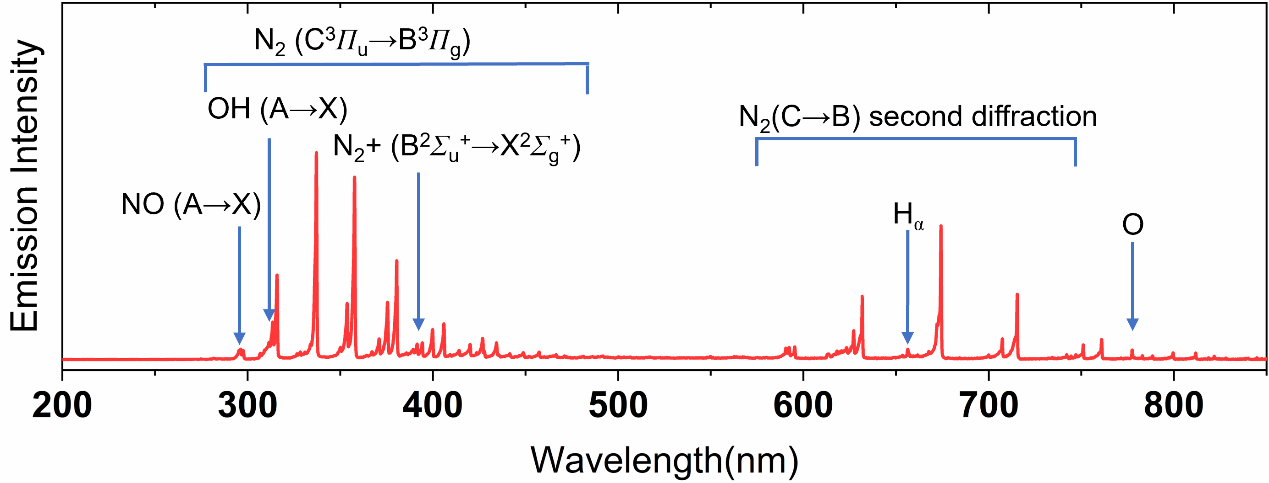


**Figure. S7** Optical emission spectra at the N_2_/O_2_ ratio of 0.8:0.2


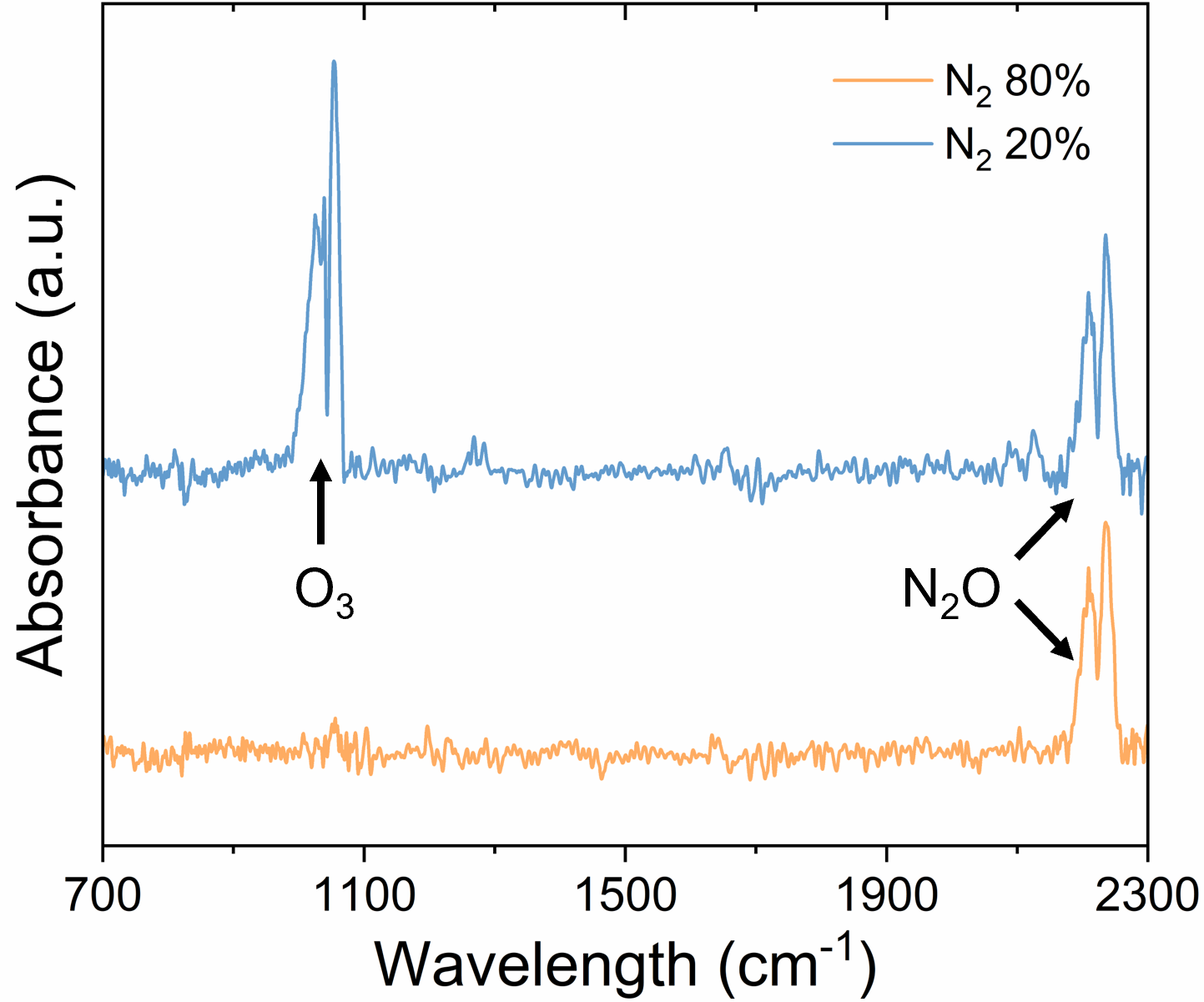


**Figure. S8** FTIR spectra at different N_2_/O_2_ ratios





**Figure. S9** Optical emission spectra at different N_2_/O_2_ ratios


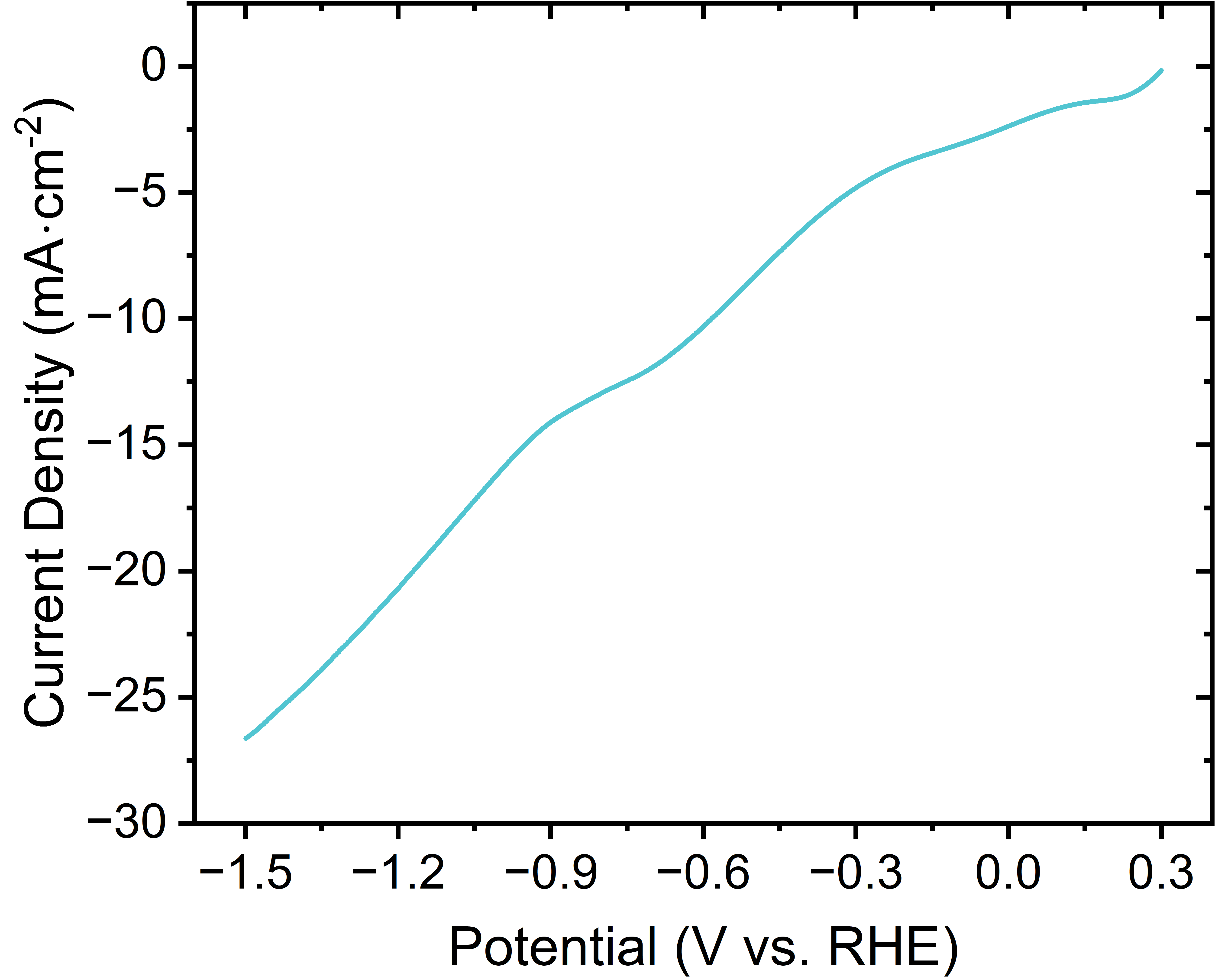


**Figure. S10** LSV curves for 2 mM NO_3_^-^ (PH=2)

**3. Plasma chemistry modelling**

**3.1 Model description and estimation of input parameters**

To comprehend the distinct characteristics of spark and DBD air discharges under various rising time conditions of applied high voltage, zero-dimensional plasma chemistry modelling was conducted. The previously established N_2_-O_2_ plasma model^[1, 2]^, implemented using the open-source ZDPlasKin^[3]^, was updated by including more high-lying vibrationally excited N_2_ species up to vibrational quantum number υ=15 states. This enhancement aimed to explore the potential significance of enhanced chemical reactions in NO molecule formation via N_2_(υ) and atomic oxygen interactions, which exhibit an activation energy threshold approximately at E_th_ ~ 3.3 eV, closely matching the excitation threshold energy of the N_2_(υ = 12) state at 3.23 eV, expected to significantly increase the chemical reaction rate for NO formation above υ = 12 state.

Different electron density profiles and reduced electric fields were tested for rising times Δt 50 ns, 200 ns, and 500 ns, to simulate varying plasma conditions based on voltage-current characteristics and optical emission observations from the iCCD.

To simulate nitrite and nitrate production in the liquid water volume, we considered different solvation properties of individual gas species and determined the input supply rates of corresponding aqueous phase species in a manner consistent with our previous studies^[1, 2]^, following the approach of Leitz and Kushner^[4]^. However, it is important to note that the water chemistry model assumes a fixed gas-water boundary and does not account for dynamic transport through numerous bubbles, which can enhance the transport of reactive species to the liquid volume and increase reaction times in the gas phase.

As introduced in section 2.8, a zero-dimensional kinetic models were prepared for gas and liquid domain using open-source ZDPlaskin.^[5]^ The gas or aqueous phase reactions considered in the kinetic input data file as shown in equation (1) are converted and merged into particle conservation equations (2) for each species i, and by solving these coupled differential equations, the temporal density profile of different species and its related reaction rate information can be provided.

$aA+bB \longrightarrow a^{'}A+cC$ (1)

$\frac{d[N_{i}]}{dt}= {\sum_{j=1}^{j_{max}} Q_{ij}}$ (2)

where, [*Ni*] is the density of species i. For example, regarding reaction *j* of species A as shown in equation (1), $Q_{Aj}=\left( a^{'}-a \right)k_{j}{[A]}^{a}{[B]}^{b}$ , $k_{j}$ is the rate coefficient of the individual reaction *j*, [A] and [B] indicates the density of species A and B, a and b are the stoichiometric coefficients in the equation (1) for species A and B respectively. The distinct discharge volumes of the DBD and spark regions were represented by different residence times in this zero-dimensional model, as depicted in Figure S11.

**
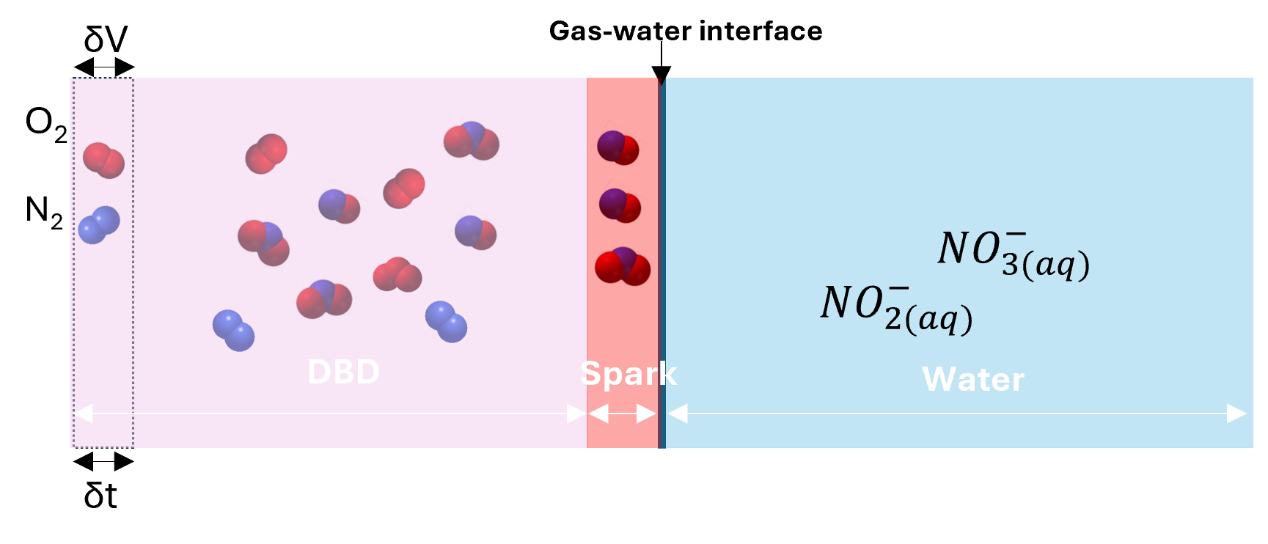
**

Figure S11. Illustration of structure of combined chemistry model in different domain of N_2_-O_2_ plasma system for NOx synthesis, where δV indicates infinitesimal volume element in spatial domain and δt is corresponding time interval in temporal domain.

The combined BOLSIG+^[6]^, which is a Boltzmann equation solver, provides the reaction coefficients related to electron interactions, such as electron attachment, excitation, ionization and dissociation. The gas-phase reactions between N_2_ and O_2_, as well as H_2_O including vibrational-vibrational and vibrational-translational interactions, were adapted from Capitelli et al.^[7]^ as summarized elsewhere. ^[8, 9]^ For the water chemistry model, most of reactions were obtained from Leitz and Kushner^[10]^ as in our previous study.^[1, 2]^

The species considered in the model are listed in Table S1, where it is noteworthy that additional high-lying N_2_(υ) species up to υ=15 were included to investigate the impact of enhanced chemical reactions involving these vibrationally excited N_2_(υ) species on NO production. The reaction rate coefficients were calculated as suggested by equation (3) from Capitelli et al. ^[7]^

$k_{\nu}\left( T \right)\left[ {cm}^{3}s^{-1} \right]={\frac{{(E_{\nu}+3000)}^{a_{1}}}{T^{a_{2}}}exp\left( a_{3}+ \frac{38370}{T}a_{4}+\frac{E_{\nu}}{T}a_{5} \right)}$ (3)

$E_{\nu}=3395\nu\left[ 1-6.217\times{10}^{-2}(\nu+1) \right]$ in Kelvin, the coefficient α_i_ are given in Table below as provided in Capitelli et al. ^[7]^

| ν | α_1_ | α_2_ | α_3_ | α_4_ | α_5_ |
| --- | --- | --- | --- | --- | --- |
| 0 ≤ν≤ 8 | -0.419312 | -0.37836 | -23.04468 | -0.992436 | 0.989385 |
| 9 ≤ν≤ 12 | -3.42306 | -1.4234 | 1.423118 | -0.919692 | 0.917323 |
| 13 ≤ν≤ 23 | 6.4805404 | -0.279371 | -96.75885 | -0.037869 | 0.019647 |

**Table S1.** Gas phase, surface adsorbed and aqueous species considered in the model ^§^where, the corresponding aqueous phase species of nitrogen, oxygen and N_x_O_y_ gas species listed above are included in the aqueous kinetic model

| **Ground-state molecules**  **and radicals** | N_2_, O_2_, O_3_, H_2_O, NO, NO_2_, NO_3_, ·OH, NO, NO_2_, NO_3_, N_2_O, N_2_O_3_, N_2_O_4_, N_2_O_5_, H_2_, HNO, HNO_2_, HNO_3_, H_2_O_2_, HO_2_ |
| --- | --- |
| **Vibrationally excited**  **molecules** | N_2_(v_i_, i=1-15), O_2_(v_i_, i=1-4), H_2_O(v_i_, i=1-3) |
| **Electronically excited molecules** | N_2_(A3), N_2_(B3), N_2_(a`1), N_2_(C3), O_2_(a1) O_2_(b1) |
| **Atoms** | N, N(2D), N(2P), O, O(1D), O(1S), H |
| **Ions** | N^+^, N_2_^+^, N_3_^+^, N_4_^+^, O^+^, O_2_^+^, NO^+^, N_2_O^+^, O^−^, O_2_^−^, H^+^ |
| **Aqueous species** | H_2_O_(aq)_, NO_2_^-^_(aq),_  NO_3_^-^_(aq),_ HO_2(aq)_, OH_(aq)_, HNO_(aq)_, HNO_2(aq)_, HNO_3(aq)_, H_2_O_2(aq)_, HO_2_NO_2(aq)_, H_3_O_(aq)_, ONOOH_(aq)_,  H_2_O^-^_(aq)_, H_2_O^+^_(aq)_, HO_2_^−^_(aq)_, OH^−^_(aq)_, O_2_NO_2_^−^_(aq)_, H_3_O^+^_(aq)_ ^§^ |

To characterize the different plasma conditions at various rising times of applied high voltage, input parameters such as reduced electric field and electron densities for modelling were selected to best match the measured peak current and power. The estimated discharge volumes for DBD and Spark were 0.817 cm³ and 6.28 × 10⁻² cm³, respectively. Considering a flow rate of 1 LPM, the average residence times of molecules were taken as 50 ms and 3.71 ms in DBD and Spark, respectively. However, it is important to note that estimating gas discharge volumes, especially in highly dynamic and transient spark discharges, inherently involves large errors, as discussed in our previous study^[2]^.


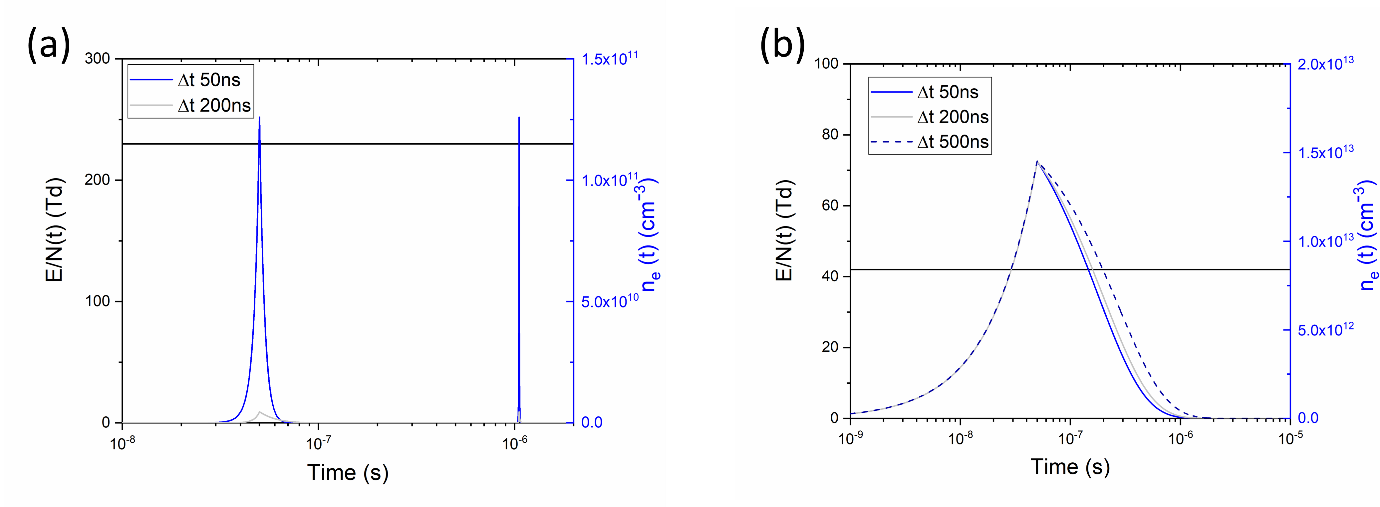


**Figure S12.** Temporal profile of input parameters with different rising time: (a) for DBD region and (b) for Spark region

**Table S2.** Estimated input parameters used for the model based on the characteristic dimension and time scale and measured peak current and power density for Spark and DBD

|  | E/N  (Td) | Max. n_e_  (cm^-3^) | Time constant*  (s) | Gas temperature  (K) | Average power  (W) | Peak current  (A) | Peak power  (kW) |
| --- | --- | --- | --- | --- | --- | --- | --- |
| Rising time Δt 50ns | | | | | | | |
| DBD | 230 | 1.26×10^11^ | 2×3×10^-9^ | 350(assumed) | 1.1 | 3.6(model) | 17.5(model) |
|  |  |  |  |  |  | 4.9 (meas.) | 23.0 (meas.) |
| Spark | 42 | 1.45×10^13^ | 1.75×10^-7^ | 400 | 5.7 | 0.94(model) | 8.3(model) |
|  |  |  |  |  |  | ~1.0(meas.) | ~8.0(meas.) |
| Rising time Δt 200ns | | | | | | | |
| DBD | 230 | 4.58×10^9^ | 2×1×10^-8^ | 300(assumed) | 0.08 | 0.13(model) | 0.75(model) |
|  |  |  |  |  |  | † | † |
| Spark | 42 | 1.45×10^13^ | 2.6×10^-7^ | 450 | 7.2 | 0.96 | 9.8 |
|  |  |  |  |  |  | ~0.8(meas.) | ~5.0(meas.) |
| Rising time Δt 500ns | | | | | | | |
| Spark | 42 | 1.45×10^13^ | 2.7×10^-7^ | 500 | 7.0 | 1.45 | 9.2 |
|  |  |  |  |  |  | ~1.0(meas.) | ~8.2(meas.) |

* The decay time constant of the exponential profile of electron density n_e_ serves as an indicator for the effective duration of electron presence. For a full single period of the high-voltage square pulse at 2.51×10⁻⁴ s (4 kHz), two instances of short n_e_ pulses were used to model DBD, while a longer-lasting single n_e_ pulse was assumed to represent spark discharge, consistent with the measurements.

†In the measured current profile, high current of short duration was observed even under Δt 200 ns and Δt 500 ns conditions. However, this was primarily attributed to the displacement current component rather than the conduction current from free charges in the plasma. This interpretation is further supported by the significantly weak optical emission intensity observed with the iCCD. Therefore, the significantly low current, and hence the assumption of low electron density, was considered reasonable for characterizing a possible DBD region at Δt 200 ns condition.

For the DBD region, an initial gas composition of N_2_:O_2_ = 0.8:0.2 was assumed, and for subsequent spark regions for Δt 50 ns and 200 ns condition, the output densities from the developed chemistry in DBD during estimated residence times were taken as initial gas compositions. Near the spark-water interface during the final electron pulse, 0.5 vol% H_2_O was introduced to account for the influence of water presence, considering potential strong spatial gradients. The impact of introduced H_2_O near the bubble boundary in the spark discharge region is further discussed in the SI with reference to Figures S15 and S16.

**3.2 Comparison of vibrationally excited N_2_(υ) species at different rising time condition**

To confirm the experimental observation on the characteristic vibrational temperatures at different plasma discharge condition, we compared the density and distribution of vibrationally excited N₂(υ) species under different rise time conditions, as shown in Fig. S14. In Fig. S14(a), for the Δt = 500 ns condition, as summarized in Table S2, a rapid increase in N₂(υ) density was confirmed under higher power and longer-lasting electron pulses at high gas temperatures, achieving a comparable level to that of the Δt = 50 ns condition which enabled a coupled DBD and spark discharge. However, in the plasma discharge with Δt = 50 ns, due to the contribution of vibrationally excited species from the DBD discharge, as well as reactive species including various forms of NOx, the total N₂(υ) density was 1.12 times higher (3.2×10^18^ cm^-3^) than under the 500 ns condition (2.8×10^18^ cm^-3^) at the peak electron density of the last applied voltage pulse period. An abrupt decrease in total N_2_(υ) density is noticed at the final voltage pulse and it is due to the strong V-T relaxation of N_2_(υ) by H_2_O molecules. It is thought to be important reason to decrease NOx production, as briefly discussed in section 3.1.2.

The vibrational distribution function, plotted in Fig. S14(b), shows a higher ratio of high-lying N_2_(υ), which indicates higher vibrational temperature (T_vib_) under the Δt = 50 ns condition compared to Δt = 500 ns condition, especially when the electron density pulse decays. This is considered to be due to the higher V-V interaction rate resulting from the higher density of N₂(υᵢ) species in Δt = 50 ns condition as Davies et al.^[11]^ investigated on the importance of vibrational energy transfer processes.

**
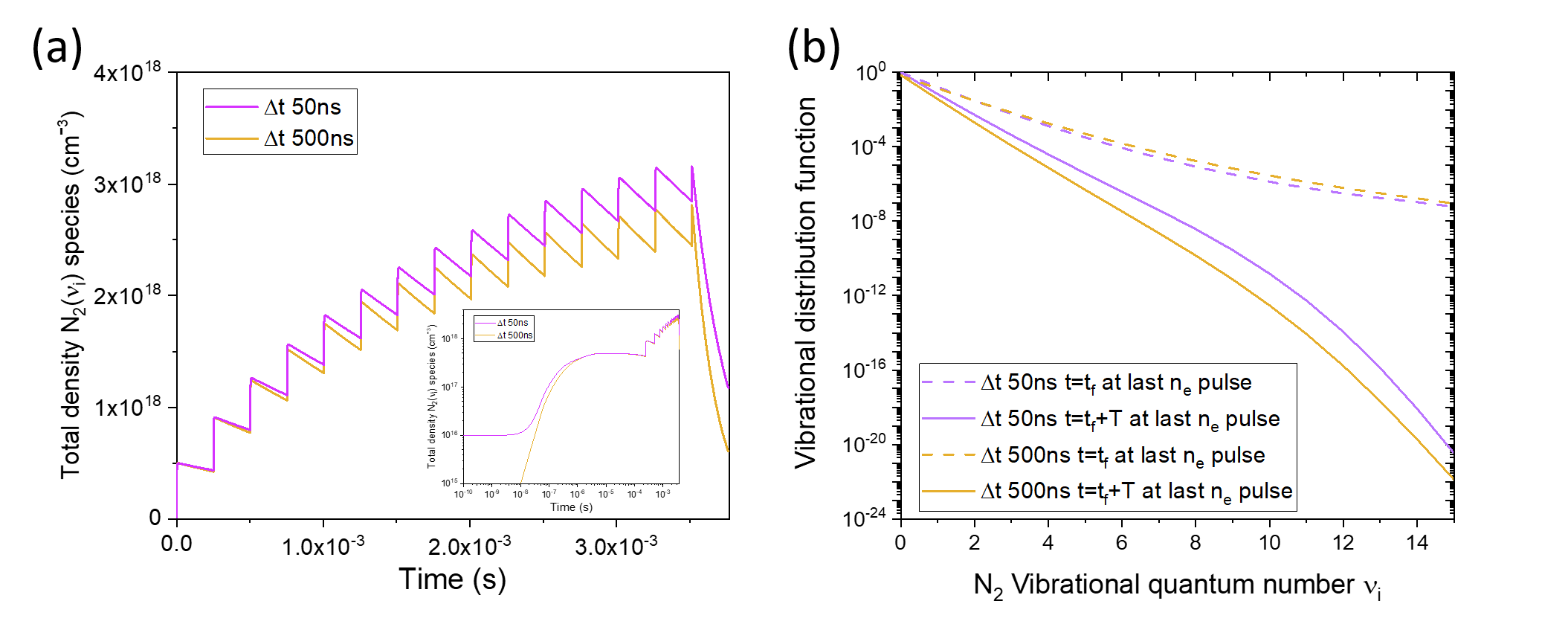
**

**Figure S13** Comparison of (a) total N₂(υᵢ) species density and (b) vibrational distribution function of N₂(υ) in the spark discharge region under the Δt = 50 ns and 500 ns condition. Here, *t_f_* indicates the time at the beginning of the last electron pulse (t = 3.5 × 10⁻³ s) in Fig. S13(a), and *T* represents the total time period of the input voltage (2.51 × 10⁻⁴ s). The inset highlights the differences in N₂(υ) densities between the Δt = 50 ns and Δt = 500 ns conditions, particularly at the initial stage of the spark discharge.

**3.3 NOx production in spark only discharge at Δt 500ns condition**

Figure S14 depicts the density profile of key species in the N_2_-O_2_ gas-phase discharge under Δt 500 ns conditions, with input parameters estimated as suggested in Section 1 of the Supplementary Information. At gas temperature 500K in spark-only discharge for Δt 500 ns— the highest temperature among all tested conditions— the difference between NO and NO_2_ densities increases, and the dissociation of N_2_O_y_ species accelerates, diminishing its contribution compared to Δt 50 ns conditions. During the final electron pulse just before reaching the gas-water interface, NOx levels decrease due to interaction with H_2_O molecules, similar to the Δt 50 ns case, mostly converting into highly soluble HNOx, which contributes to the formation of NO_2(aq)_^-^ and NO_3(aq)_^-^ in water.

Ozone remains at a high number density in this gas discharge model primarily due to the relatively moderate gas temperature. However, at the water interface, ozone solvation is limited, and its converted aqueous-phase ozone can be extensively utilized in water reactions. This may explain the significantly lower ozone concentration observed in measurements compared to simulations in the gas-phase discharge model.





**Figure S14** Density profile of important species in N_2_-O_2_ spark discharge at rising time Δt 500 ns condition

**3.4 Comparison of different production reaction for NO**

To gain a better understanding of the important mechanisms in NOx production, we compared the contribution from different NO production reactions in DBD and spark conditions at Δt 50 ns and Δt 500 ns, as shown in Fig. S15-S16. In the DBD region, depicted in Fig. S15(a), the O_2_ + N reaction, including the contribution from O_2_(υ), was found to play a critical role, especially in the early stages of residence time. As the density of N_2_O increases, the reaction between N_2_(A) and N_2_O becomes more significant. Additionally, with increasing NO_2_ density over time, the dissociation of NO_2_ by atomic O or N appeared to be an important pathway for maintaining NO density in the discharge.

In contrast, during spark discharge, the contribution of N_2_(υ) + O interaction shows a rapid increase due to the presence of high-lying N_2_(υ) species in the high-density spark discharge region.

An abrupt decrease in N₂(υ) + O interactions during the final pulse of the spark discharge is observed under both Δt = 50 ns and Δt = 500 ns conditions, similar to the decrease in the high-lying N₂(υ) distribution. This decrease is attributed to the strong V-T relaxation of N₂(υ) caused by H₂O molecules, as discussed earlier. This phenomenon is thought to be the main reason for the decreased NOx density and the conversion into HNOx.


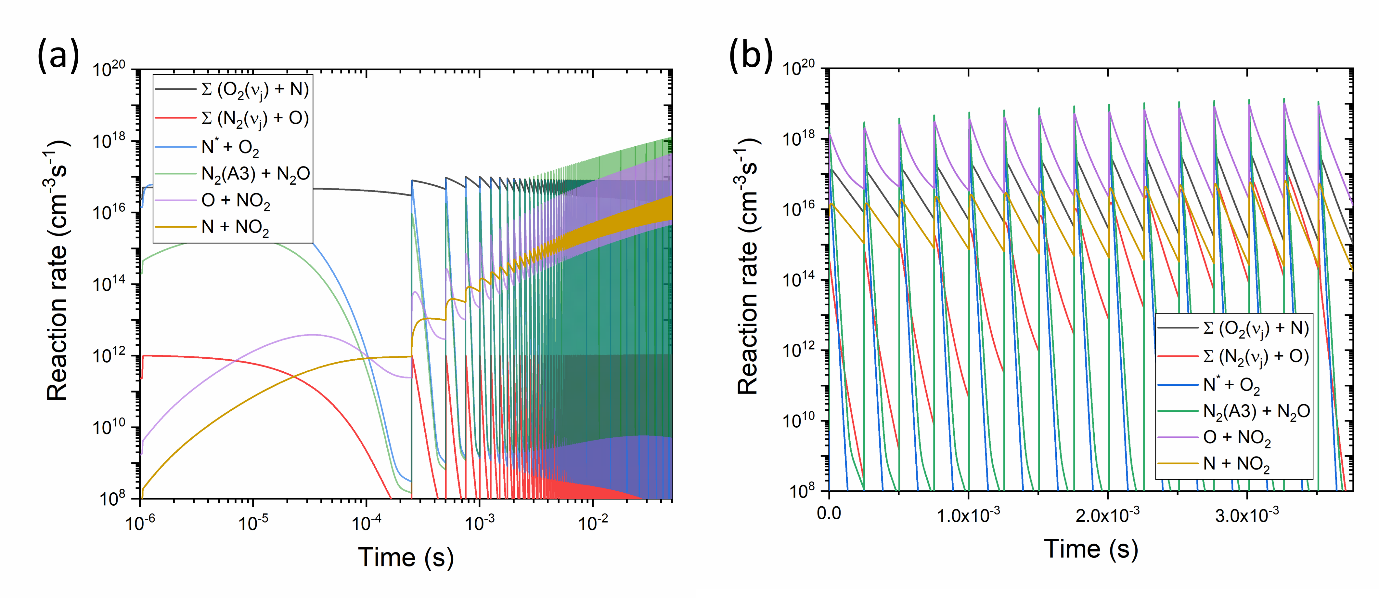


**Figure S15** Comparison of important reactions for production of NO in (a) DBD and (b) following spark discharge region at rising time Δt 50ns condition


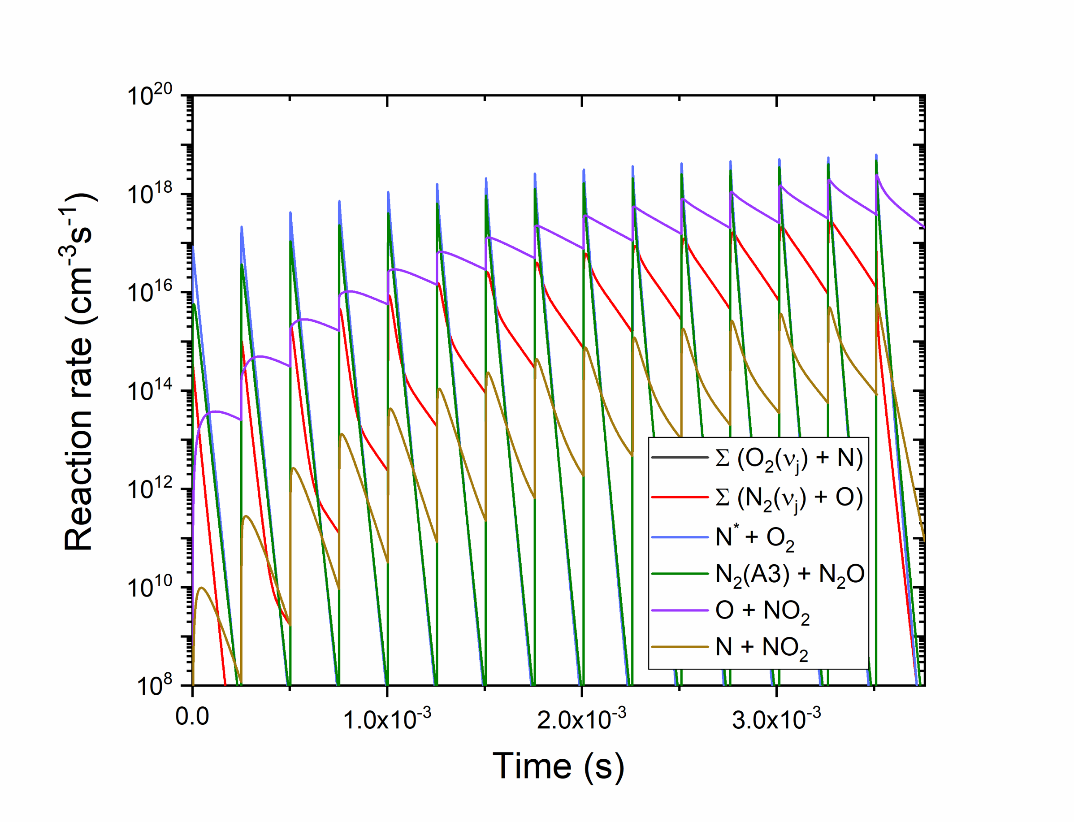


**Figure S16** Important production reactions of NO in spark only discharge at Δt 500ns condition

**3.5 Different composition of NOx species in gas and water at different rising time conditions**

Figure S17 illustrates the different production rates and compositions of NOx species under different rising time conditions. With combined DBD and spark discharge at Δt 50 ns, it sustains a comparable amount of NO and NO_2_ in the discharge volume, along with converted HNOx from interactions with H_2_O. In contrast, using spark discharge alone at Δt 500 ns significantly increases the production rate of NO compared to other NOx species.

In terms of water chemistry, nitrate appears to be the predominant form of dissolved NOx species under both conditions within the specified operating range, as observed in experiments. As shown in Fig. S18, O_2(aq)_^-^ and NO_(aq)_ contribute most significantly to NO_3(aq)_^-^ formation, followed by interactions between NO_2(aq)_ and H_2_O_(aq)_. Both NO_(aq)_ and NO_2(aq)_ play important roles in producing NO_2(aq)_^-^ as well, interacting with OH_(aq)_ and H_2_O_(aq)_ respectively, as depicted in Fig. S18(a). The contributions from HNO_3(aq)_ and HNO_2(aq)_ to the formation of NO_3(aq)_^-^ and NO_2(aq)_^-^ are also noteworthy.

However, the model only suggested a significantly lower absolute production rate of NOx, with an opposite trend observed in the relative ratio of NO_2(aq)_^–^ / NO_3(aq)_^–^, which indicates limitations in satisfactorily explaining the experimental measurements. It is important to stress again the possible errors in estimating discharge volume for the spark region and the inadequacy of zero-D modelling to convey dynamic transport properties through the bubble interface.


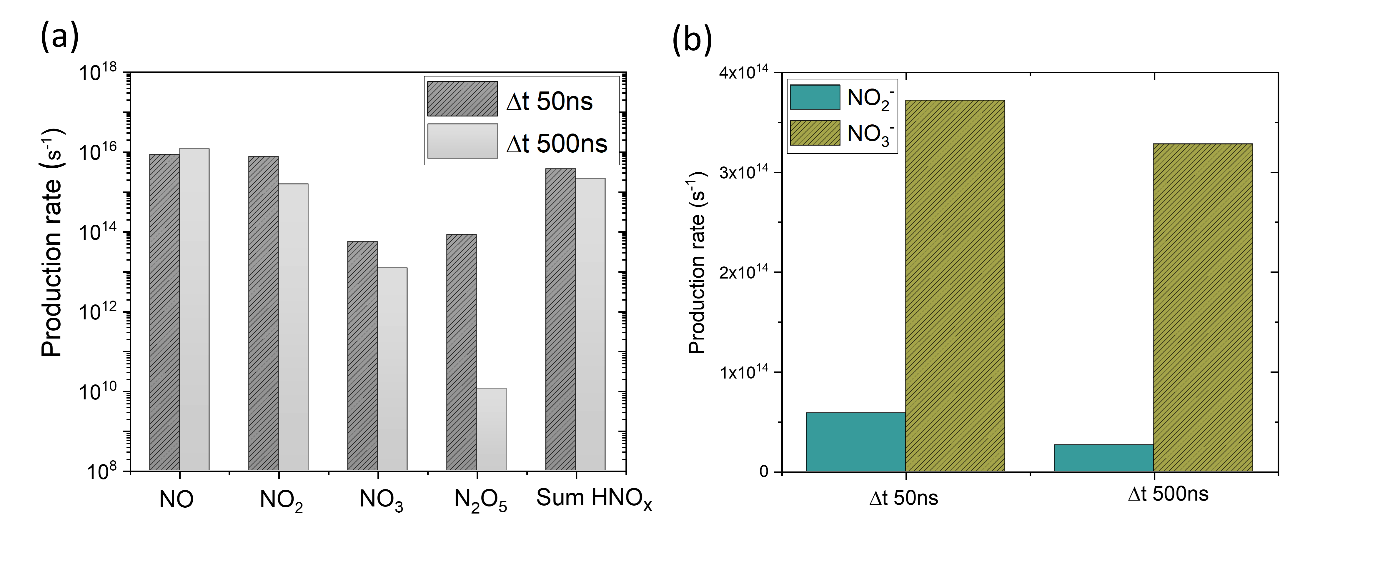


**Figure S17** Comparison of production rate of important NOx species (a) in gas phase and (b) in water volume


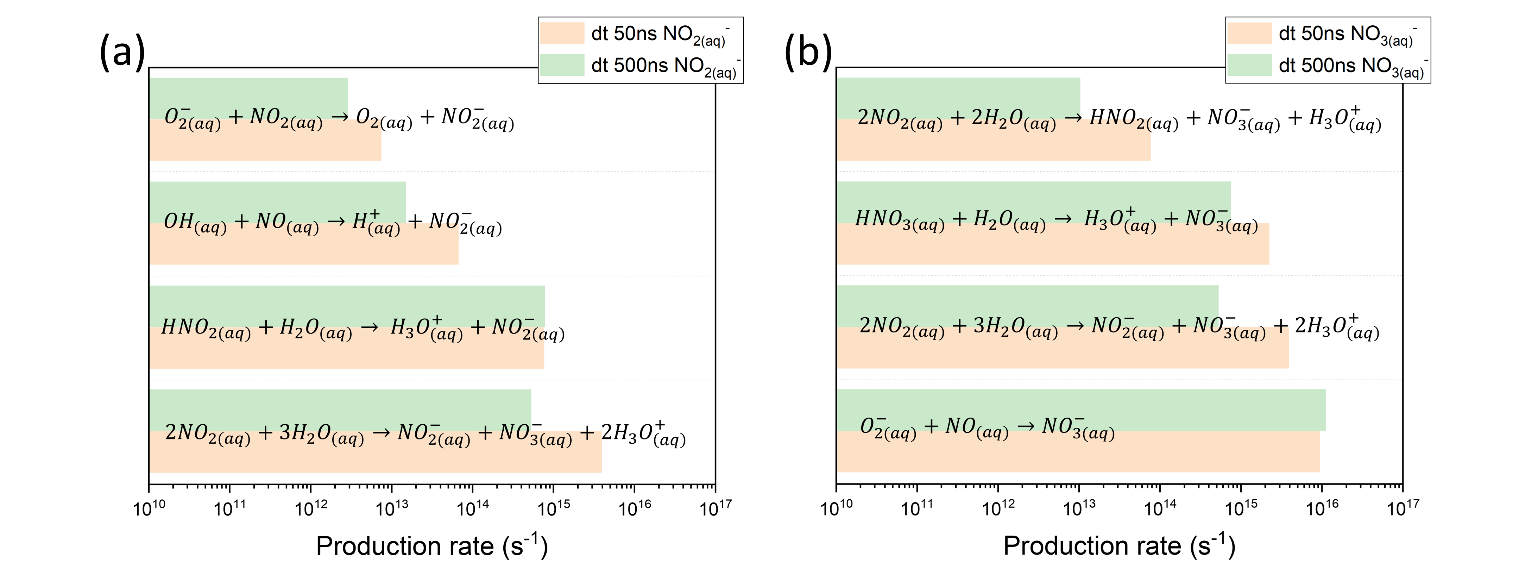


**Figure S18** Comparison of important production reactions for NO_2(aq)_^-^ and NO_3(aq)_^-^ in water at rising time Δt 50 ns and 500 ns condition

**4. Planting Applications**

We prepared a commercial ammonium nitrate solution with the same concentration as the experimental solution and conducted a comparative plant growth experiment. Photographs of the three groups of cabbage, along with stem length and leaf length statistics, are presented in Figures S19, S20, and S21.

The images show that compared to the untreated group, the cabbages fertilized with both the experimental and commercial ammonium nitrate solutions exhibited more vigorous leaf growth and longer stems. By Day 12, the untreated cabbages exhibited blackening and necrosis at the leaf edges, while the fertilized groups remained healthy. Additionally, there was no significant difference in growth between the group treated with the commercial fertilizer and the group treated with the experimental fertilizer.

Figures S20 and S21 illustrate the changes in stem and leaf length over time for the three groups. The results indicate that the cabbages treated with the experimental ammonium nitrate solution showed no significant difference in stem or leaf growth compared to those treated with the commercial ammonium nitrate solution. Both groups displayed significantly greater stem and leaf lengths than the untreated group.


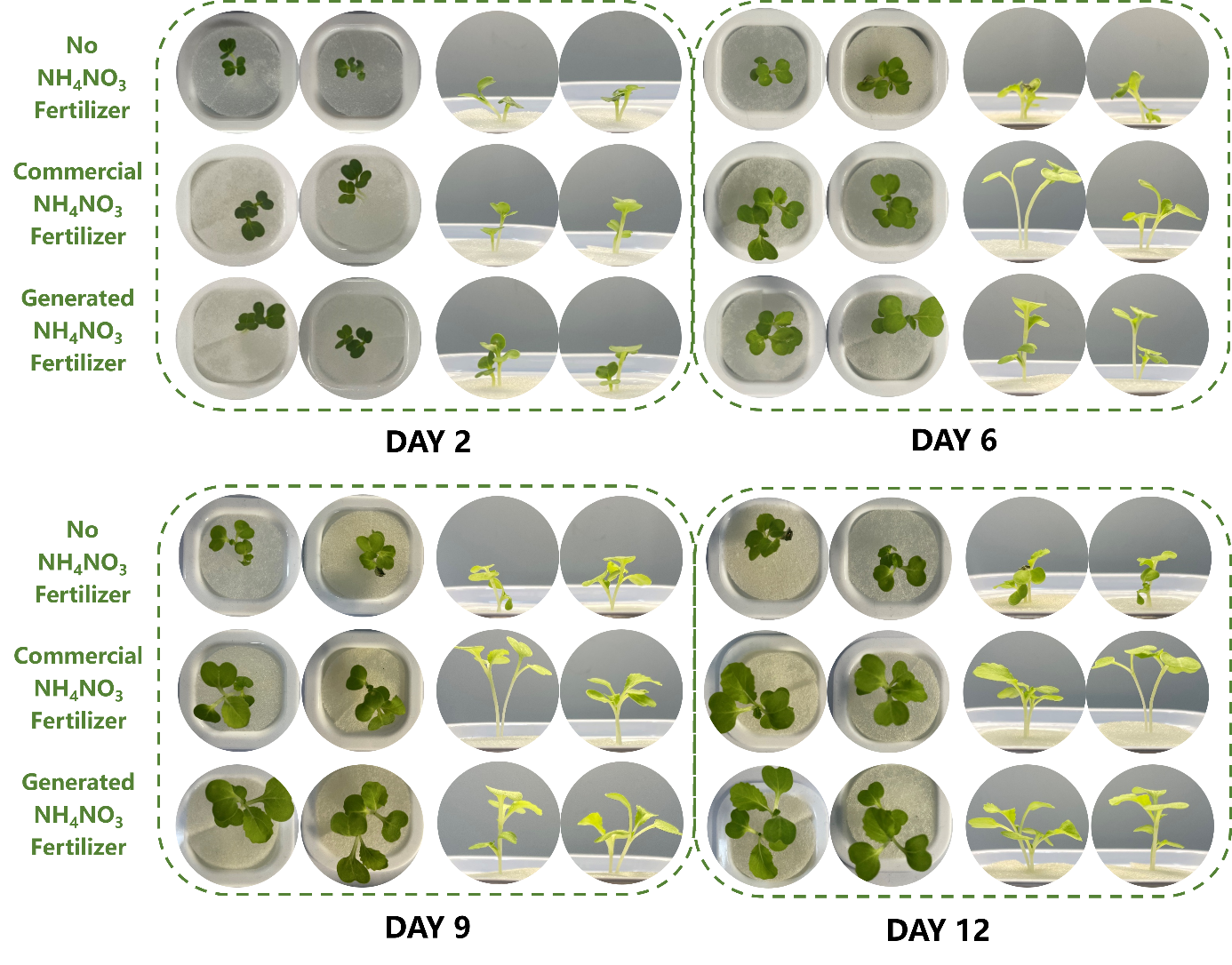


**Figure S19**. Comparison of growth state between commercial fertilizer-applied plants, generated fertilizer-applied plants and controls.


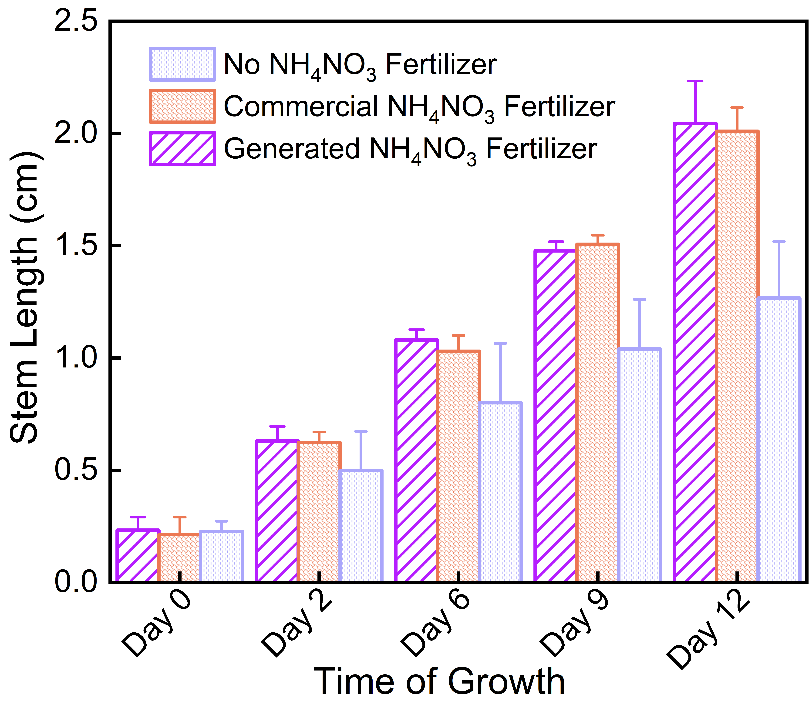


**Figure S20.** Comparison of stem length.


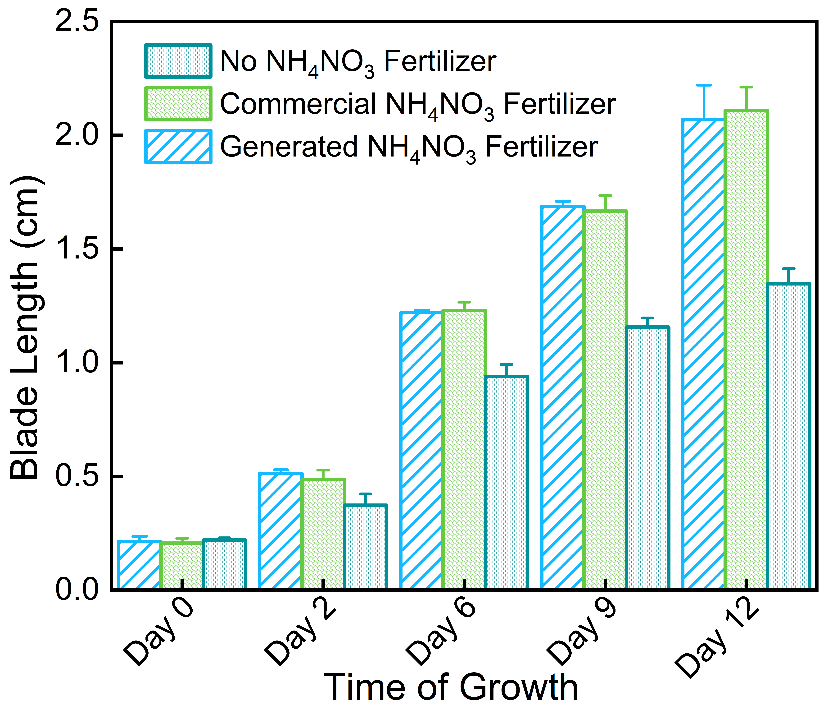


**Figure S21.** Comparison of blade length.

**5. Cost Estimation**

Assuming this technology produces 100,000,000 L of 1 mM liquid ammonium nitrate fertilizer annually, with a capacity factor of 0.8, the daily production volume of ammonium nitrate solution is estimated to be 342,465.75 L.

**Raw Material Costs:** This technology directly utilizes air and water. Air incurs no cost, but due to the high water consumption for liquid-state fertilizer, approximately 343 tons of water are used daily. The cost of agricultural irrigation water in China is about $0.0015 per ton, leading to a daily water cost of $0.51.

**Energy Costs:** The amount of NOx required for plasma generation is 684.93 mol. In this experiment, using air and water as raw materials, with a pulse repetition frequency of 4 kHz and pulse rise/fall times of 50 ns, the energy consumption is 25.3 MJ/mol. Assuming energy consumption remains constant in mass production, the daily electricity requirement for plasma nitrogen fixation is 4813.53 kWh.

During electrocatalytic reduction, a voltage of 0.7 V is applied, and the current is calculated based on Faradaic efficiency, with a total electron transfer of 2.6385 × 10⁸ C, leading to an electrocatalytic energy consumption of 51.30 kWh.

Electricity prices for industrial use vary across regions in China, with the lowest at $0.05/kWh and the highest at $0.077/kWh. For this analysis, we assume an electricity cost of $0.065/kWh. This results in an average daily plasma electricity cost of $312.84 and an electrocatalytic electricity cost of $3.33.

**Equipment and Maintenance Costs:** Plasma generator: $260,000; Pulse power supply: $500,000; Electrochemical H-cell: $10,000; Electrocatalytic foam copper catalyst: $40,000; Electrochemical workstation: $300,000. Assuming a 10-year lifespan, the average daily material cost is $304.11.

Assuming maintenance costs are 10% of the total equipment cost, the average daily maintenance cost is $30.4.

Assuming depreciation costs are 7% of the total equipment cost, the average daily depreciation cost is $21.3.

**Labor Costs:** Given the small scale of this production line, labor costs are assumed to account for 5% of the total costs, or $35 per day.

**Reference**

[1] J. Sun, T. Zhang, J. Hong, R. Zhou, H. Masood, R. Zhou, A. B. Murphy, K. K. Ostrikov, P. J. Cullen, E. C. Lovell, R. Amal, A. R. Jalili, *Chem. Eng. J.* **2023**, *469*, 143841, <https://doi.org/10.1016/j.cej.2023.143841>.

[2] J. Sun, R. Zhou, J. Hong, Y. Gao, Z. Qu, Z. Liu, D. Liu, T. Zhang, R. Zhou, K. Ostrikov, P. Cullen, E. C. Lovell, R. Amal, A. R. Jalili, *Appl. Catal., B* **2024**, *342*, 123426, <https://doi.org/10.1016/j.apcatb.2023.123426>.

[3] S. Pancheshnyi, B. Eismann, G. Hagelaar, L. Pitchford, *Bulletin of the American Physical Society* **2008**.

[4] A. M. Lietz, M. J. Kushner, *J. Phys. D: Appl. Phys.* **2016**, *49* (42), 425204, <https://doi.org/10.1088/0022-3727/49/42/425204>.

[5] S. Pancheshnyi, B. Eismann, G. Hagelaar, L. Pitchford, *University of Toulouse, LAPLACE, CNRS-UPS-INP, Toulouse, France* **2008**.

[6] G. J. M. Hagelaar, L. C. Pitchford, *Plasma Sources Sci. Technol.* **2005**, *14* (4), 722, <https://doi.org/10.1088/0963-0252/14/4/011>.

[7] M. Capitelli, C. M. Ferreira, B. F. Gordiets, A. I. Osipov, *Plasma Physics and Controlled Fusion* **2000**, *43*, 371.

[8] J. Hong, T. Zhang, R. Zhou, L. Dou, S. Zhang, R. Zhou, B. Ashford, T. Shao, A. B. Murphy, K. Ostrikov, P. J. Cullen, *Green Chem.* **2022**, *24* (19), 7458, <https://doi.org/10.1039/D2GC02299K>.

[9] J. Hong, S. Pancheshnyi, E. Tam, J. J. Lowke, S. Prawer, A. B. Murphy, *Journal of Physics D: Applied Physics* **2017**, *50* (15), 154005.

[10] A. M. Lietz, M. J. Kushner, *Journal of Physics D: Applied Physics* **2017**, *50* (11), 119501, <https://doi.org/10.1088/1361-6463/aa5c2e>.

[11] H. L. Davies, V. Guerra, M. van der Woude, T. Gans, D. O’Connell, A. R. Gibson, *Plasma Sources Science and Technology* **2023**, *32* (1), 014003, <https://doi.org/10.1088/1361-6595/aca9f4>.
